# Development of digital hardware for a spiking image recognition network employing a novel burst-based reinforcement learning approach

**DOI:** 10.1038/s41598-025-28901-x

**Published:** 2025-12-30

**Authors:** Soheila Nazari, Masoud Amiri

**Affiliations:** 1 Faculty of Electrical Engineering, Shahid Beheshti University, Tehran, 1983969411 Iran; 2 Department of Biomedical Engineering, School of Medicine, Kermanshah University of Medical Sciences, Kermanshah, Iran; 3 Medical Technology Research Center, Institute of Health Technology, Kermanshah University of Medical Sciences, Kermanshah, Iran

**Keywords:** Reinforcement burst time dependent plasticity (RBTDP), Linear substitution technique, Hardware design, High accuracy and fast learning method, Spiking vision network, Computational biology and bioinformatics, Engineering, Mathematics and computing, Neuroscience

## Abstract

The primary focus of accurate and cost-effective computation in machines endowed with advanced cognitive abilities is to enhance the accuracy and speed of learning in the bio-inspired spiking machine vision networks. This paper introduces a novel reinforcement burst time dependent plasticity (RBTDP) learning algorithm, implemented as a digital circuit within a spiking network that utilizes low-cost neuron circuits. This paper introduces an efficient hardware solution that employs linear substitution technique, motivated by the need for precise and fast calculations that minimize costly resource consumption in machine vision platforms, particularly those utilizing neural networks. The suggested digital designs, emphasizing the linear substitution method within digital learning and neuron blocks, are meticulously detailed to ensure maximum speed enhancement, minimal resource utilization, and high accuracy. The suggested digital learning mechanism and neuron modules were employed to build a bio-inspired spiking vision network consisting of three layers and Actor and Critic neural population, which supports unsupervised and reinforcement training, utilizing excitatory AMPA and inhibitory GABA neural interactions. Consequently, the suggested bio-inspired spiking network, utilizing the proposed RBTDP learning method, demonstrated exceptional performance in spiking vision networks. Upon training on the MNIST, CIFAR10, and CIFAR100 datasets for 6, 6, and 30 training iterations respectively, the model achieved remarkable accuracies of 98.2%, 94%, and 75.6%. These results reflect both enhanced accuracy and faster convergence compared to earlier studies.

## **Introduction**

Considering that intelligent machines require complex and extended training processes, it is crucial to focus on the hardware design of neural networks and their learning methodologies, emphasizing cost-effectiveness and energy efficiency^1^. The nervous system expends minimal energy when engaged in complex cognitive tasks^2^. Consequently, it seems that designing artificial intelligence systems that incorporate learning elements inspired by the biological findings related to the brain could be an effective approach to developing brain like hardware^3^ which is follow in this paper. On the other hand, neuromorphic system design, which may serve as a practical foundation for implementing these networks, have garnered significant attention^4,5^. Therefore, the creation of a hardware spiking vision network characterized by sparse spike activity is a significant topic addressed here.

Evidently, the brain demonstrates the greatest capacity for cognitive abilities, including learning. Spiking networks aim to mimic the computational principles of the brain to replicate this remarkable capability in intelligent machines^6^. In the field of machine applications^7–11^, deep convolutional networks, which have a more substantial research foundation, coexist with spiking neural networks. The primary benefit of spiking platforms over their deep counterparts is their asynchronous training methodology^12^. This feature significantly lowers power consumption when applied to hardware, although deep networks continue to benefit from superior learning accuracy^13^. Conversely, spiking networks are thought to provide advantages over deep networks, particularly in terms of their capacity for unsupervised learning and quicker learning^14^, as they can achieve convergence with fewer training epochs. Therefore, this study primarily focuses on the development of the brain inspired spiking vision network and the spike-based training methodologies.

The input layer of the created spiking vision network is based on a pseudo-retinal model. The second layer consists of a group of spiking Hindmarsh and Rose (HR) neurons, which include both pyramidal and interneurons. These neurons are interconnected through excitatory and inhibitory synapses, utilizing a dynamic model of AMPA and GABA neurotransmitters. To train the bio-inspired spiking vision network that has been developed, we utilize the actor and critic neural populations that facilitate reinforcement learning^15^, along with the Burst time-dependent plasticity (BTDP) method^16^, collectively referred to as the reinforcement BTDP (RBTDP) learning approach. The occurrence of spike bursts is a common pattern observed in the neuronal signaling within the nervous system. This burst activity is believed to offer enhanced information coding capabilities in comparison to individual spikes^17^ which, could be the reason why the proposed network is powerful in classifying patterns.

To this day, a variety of spiking networks have been utilized in machine vision application^18,19^. Spiking networks, although limited by their developmental stage, incorporate more advanced unsupervised training methods compared to the various methods of training and the structural designs of networks described in second generation neural network. As a result, recent research has aimed to leverage insights obtained from training methodologies and the architectural frameworks of deep vision networks to advance the techniques used in spiking networks^20^. The process of back-propagating errors is a widely recognized method in method of supervised learning, particularly prevalent in deep neural networks. This technique was first presented in the work of Lee and his associates^21^, focusing on error back-propagation in spiking networks. In the field of machine vision, deep spiking vision networks are considered the most efficient spiking networks, largely owing to the supervised spiking error back-propagation learning technique. It is essential to emphasize that the proposed RBTDP learning strategy could surpass deep spiking vision networks regarding both accuracy and learning speed.

To effectively assess the performance of the proposed networks, it is essential to utilize datasets that have been referenced in prior studies. The widely recognized datasets MNIST, which contains handwritten digits, along with CIFAR10 and CIFAR100, which consist of natural images, have been employed to assess the performance of the spiking vision network and the proposed RBTDP learning method. The MNIST dataset, due to the recent advancements in published research utilizing supervised deep learning techniques, is now considered to be a less challenging dataset^22^. Also, the CIFAR datasets have garnered increased attention in current research. Consequently, an analysis of the CIFAR datasets has been conducted to deliver a more precise assessment of the performance of the proposed spiking vision network.

This research further highlights the digital hardware design of the RBTDP module and HR neurons through the application of the linear substitution method. The aim is to tackle significant challenges associated with the development of precise and high-performance hardware that enhances the advanced cognitive skills of intelligent platforms^23,24^. A thorough methodology was employed, encompassing error analysis and assessing the effectiveness of the proposed spiking vision network on complex datasets, to validate the effectiveness of the proposed digital circuit^25^. A contemporary method for the hardware implementation of brain-derived networks involves the design of both hardware and software to leverage the benefits of both FPGA and processor platforms^26–28^. The effective functioning of the digital RBTDP and HR neurons within the spiking vision network, along with the outcomes of the cost-efficient and energy-saving FPGA implementation, demonstrate that the digital design outperforms the previous methods. Power and exponential functions are fundamental in the computations of neural networks, particularly in spiking networks. Two primary methods for assessing these functions are the approximation method and the iterative approach^29^. While these techniques have been extensively examined in various scientific literature, this paper presents an innovative solution aimed at enhancing digital circuit performance through the optimization of exponential and power function calculations via linear substitution technique. The performance of the biologically spiking vision network was evaluated using the RBTDP training rule for software results, as well as the Digital-RBTDP and digital neuron circuits for hardware results, specifically in the classification tasks involving MNIST, CIFAR10, and CIFAR100 datasets. Ultimately, the findings on classification accuracy and speed are presented, highlighting the promising advantages of this innovative approach in enhancing the precision and effectiveness of hardware, thereby enhancing the cognitive skills of intelligent vision machines.

This paper represents a noteworthy amalgamation of progress in bio-inspired computing and spiking networks. It presents a novel RBTDP learning rule for biologically spiking vision network, which outperforms earlier machine vision platforms. Additionally, this research introduces a precise approximation method for non-linear functions, facilitating the effective digital design of the RBTDP and HR neuron digital circuits within extensive spiking vision networks. The comprehensive strategy presented in this paper enhances the efficiency of calculations within neuromorphic systems and simultaneously paves the way for advancements in the learning capability of spiking networks.

## Neurons and synapses

### An illustration of the computational modeling of neurons along with its hardware architecture

Hindmarsh and Rose introduced a streamlined version of HH neuron, referred to as the Hindmarsh–Rose neuron^30^. The HR neuron is a biological model capable of exhibiting the full range of dynamic behaviors characteristic of biological neurons. The equations governing the computational model of neuron are articulated in Eq. 1.

1 $$\begin{aligned}\frac{dx}{dt} & =y-a{x}^{3}+b{x}^{2}-Z+I \\ \frac{dy}{dt} & =c-d{x}^{2}-y \\ \frac{dz}{dt} & =r\left(s\right(x-q)-z) \end{aligned}$$

Membrane potential is represented by $\:x$, while the rapid current associated with the dynamics of sodium and potassium ion channels is denoted as $\:y$. The slow current related to the dynamics of calcium channels is indicated by $\:z$. Additionally, $\:I$ represents the input stimulation current, while $\:r$ serves as the controller for frequency of spike and burst. By adjusting $\:I$ and $\:r$, various spike or burst activity can be generated.

It is clear from the Eq. 1 that this neuron incorporates the nonlinear functions $\:{x}^{2}$and $\:{x}^{3}$. The presence of nonlinear terms, along with the need of multipliers, raises the cost of hardware design and complicates the feasibility of deploying extensive neural networks^31^. To address this issue, a linear model has been proposed that substitutes the terms $\:{x}^{2}$and $\:{x}^{3}$ with optimally designed linear equations. The LHR neuron enables the implementation of cost-effective hardware while fully maintaining the dynamic features of the initial model.

A best approximation search algorithm based on algorithm 1 is introduced to identify the best optimal linear fit for the approximation of non-linear terms of the HR neuron. Initially, two random points are chosen from the non-linear curve, with each point situated within the two halves of the horizontal axis’s range of variation. Subsequently, the Normalized Mean Absolute Error (NMAE) (Eq. 7) is computed to assess the difference between the $\:{x}^{2}$and $\:{x}^{3}$ curves and their corresponding linear value. Following this, distance between two Consecutive points is adjusted, resulting in a series of linear substitution equation accompanied by their respective calculation errors. Should the minimum errors achieved fall below the specified tolerance limit for errors, the best approximation search algorithm is to be terminated. Conversely, if the errors exceed this threshold, three, four, and ultimately eleven random points will be chosen, and the preceding steps will be reiterated until the error is reduced to below the established tolerance limit. The comparative analyses confirm that the method used to identify the best approximation was executed effectively, and the linear substituted model accurately reflected the characteristic of the initial model.

**Algorithm 1:**
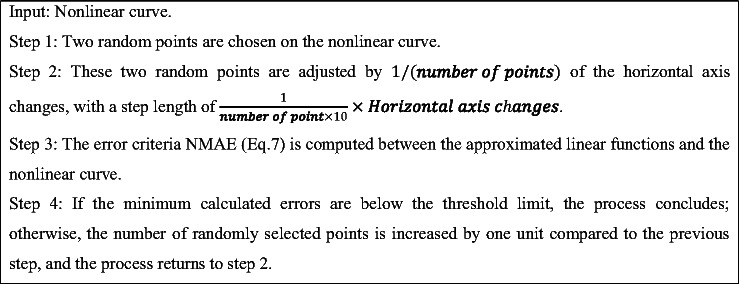
A pseudocode block of linear approximation approach.

In conclusion, the linear substituted model of $\:{x}^{2}$and $\:{x}^{3}$ is outlined as follows:

2 $$\:{x}^{3}={a}_{1}x+{b}_{1}=\left\{\begin{array}{l}6.33\:\times\:x+6.03\:\:\:\:\:if\:x<-1.3\\\:4.08\times\:x+3.1\:\:\:\:\:\:\:if-1.3<x<-1.03\\\:2.473\times\:x+1.45\:\:\:\:\:\:\:\:\:\:\:\:if-1.03<x<-0.78\\\:1.196\times\:x+0.458\:\:\:\:\:\:if-0.78<x<-0.47\:\\\:0.22\times\:x\:\:\:\:\:\:\:\:\:\:\:\:\:\:if-0.47<x\:<0\\\:0.22\times\:x\:\:\:\:\:\:\:\:\:\:\:\:\:\:\:\:if\:0<x\:<0.47\\\:1.196\times\:x-0.458\:\:\:\:\:\:\:\:\:\:\:\:\:\:\:\:\:\:if\:0.47<x<0.78\\\:2.473\times\:x-1.45\:\:\:\:\:\:\:\:\:\:\:\:\:\:\:\:\:\:\:if\:0.78<x<1.03\\\:4.08\times\:x-3.1\:\:\:\:\:\:\:\:\:\:\:\:\:\:\:\:if\:1.03<x<1.3\\\:6.33\:\times\:x-6.03\:\:\:\:\:\:\:\:\:\:\:\:\:\:\:\:\:\:\:\:\:\:\:\:\:\:\:if\:\:x>1.3\end{array}\right.$$

3 $$\:{x}^{2}={a}_{2}x+{b}_{2}=\left\{\begin{array}{l}-2.859\:\times\:x-1.997\:\:\:\:\:if\:x<-1.21\\\:-2.029\times\:x-0.99\:\:\:\:\:\:\:if-1.21<x<-0.82\\\:-1.23\times\:x-0.336\:\:\:\:\:\:\:\:\:\:\:\:if-0.82<x<-0.41\\\:-0.41\times\:x\:\:\:\:\:\:\:if-0.41<x<0\:\\\:0.41\times\:x\:\:\:\:\:\:\:\:\:\:\:\:\:\:if\:0<x\:<0.41\\\:1.23\times\:x-0.336\:\:\:\:\:\:\:\:\:\:\:\:\:\:if\:0.41<x\:<0.82\\\:2.029\times\:x-0.99\:\:\:\:\:\:\:\:\:\:\:\:\:\:\:\:\:\:if\:0.82<x<1.21\\\:2.859\:\times\:x-1.997\:\:\:\:\:\:\:\:\:\:\:\:\:\:\:\:\:\:\:\:\:\:\:\:\:\:\:if\:\:x>29.1\end{array}\right.$$

Therefore, the Linear HR neuron (LHR) (Eq. 4), which can be effectively implemented in hardware, is expressed as follows:

4 $$\begin{aligned} \frac{dx}{dt} & =y-a({a}_{1}x+{b}_{1})+b({a}_{2}x+{b}_{2})-Z+I \\ \frac{dy}{dt} & =c-d({a}_{2}x+{b}_{2})-y \\ \frac{dz}{dt} & =r\left(s\right(x-q)-z) \end{aligned}$$

**Fig. 1:**
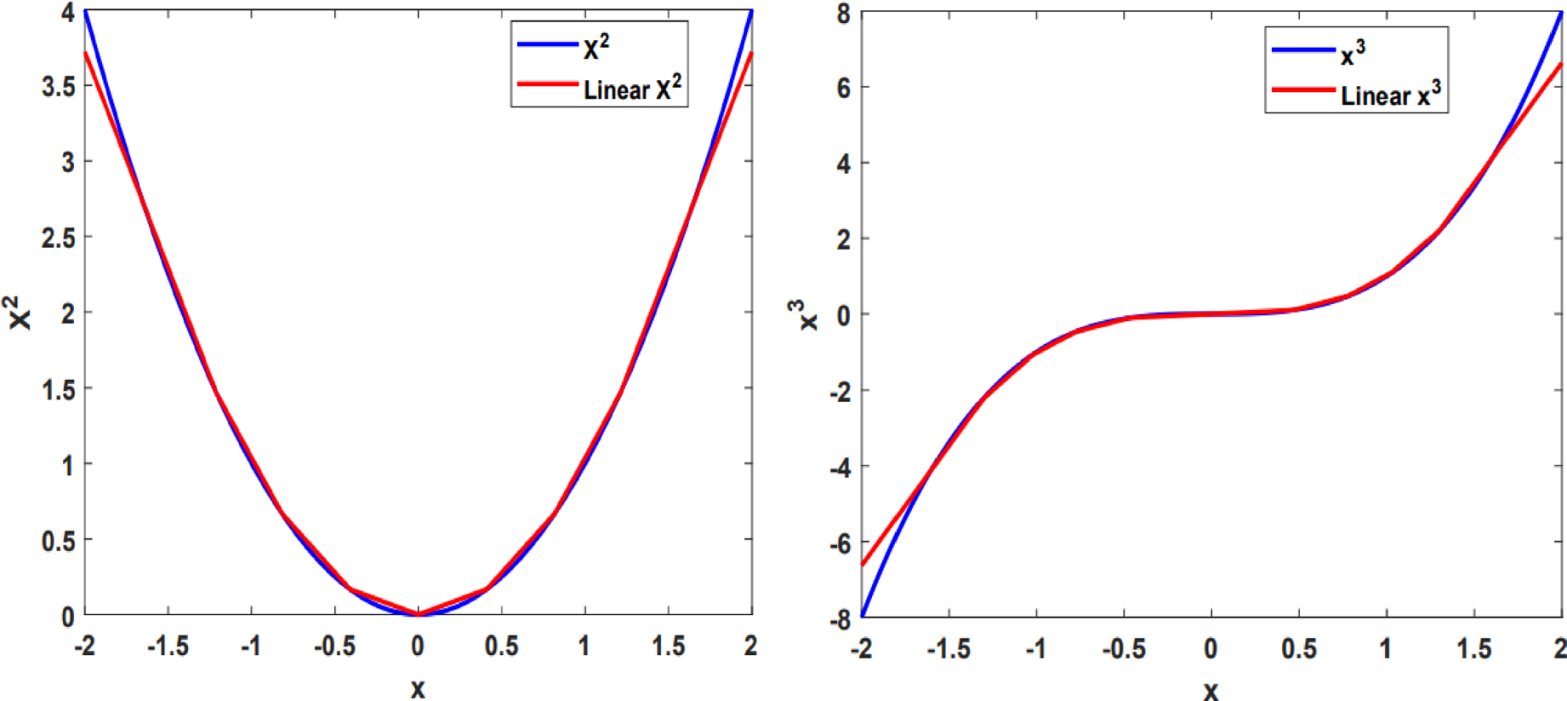
Linear approximations employed to simplify nonlinear components in the HR neuron. The blue line represents the nonlinear curve, while the red line denotes its corresponding linear approximation.

Figure 1 illustrates the linear substitution process derived from Eqs. 2–3. This linear substitution process employs the proposed search algorithm, balancing the number of lines with the associated error.

Equations 5 and 6 define the error metrics NMAE and NRMSE, which are utilized to assess the discrepancies between $\:{x}^{2}$and $\:{x}^{3}$ curves and their corresponding linear value. The computed errors are detailed in Table 1.

5 $$\:\text{N}\text{o}\text{r}\text{m}\text{a}\text{l}\text{i}\text{z}\text{e}\text{d}\:\text{M}\text{e}\text{a}\text{n}\:\text{A}\text{b}\text{s}\text{o}\text{l}\text{u}\text{t}\text{e}\:\text{E}\text{r}\text{r}\text{o}\text{r}\:\left(\text{N}\text{M}\text{A}\text{E}\right)=\frac{1}{nMax\left({R}_{Nonlinear\:Curve}\right)}\sum\:_{i=1}^{n}\left|{R}_{Nonlinear\:Curve}-{R}_{Lnear\:Curve}\right|$$

6 $$\:\text{N}\text{o}\text{r}\text{m}\text{a}\text{l}\text{i}\text{z}\text{e}\text{d}\:\text{R}\text{o}\text{o}\text{t}\:\text{M}\text{e}\text{a}\text{n}\:\text{S}\text{q}\text{u}\text{a}\text{r}\text{e}\:\text{E}\text{r}\text{r}\text{o}\text{r}\:\left(\text{N}\text{R}\text{M}\text{S}\text{E}\right)=\frac{1}{Max\left({R}_{Nonlinear\:Curve}\right)}\sqrt{\frac{\sum\:_{i=1}^{n}{({R}_{Nonlinear\:Curve}-{R}_{Lnear\:Curve})}^{2}}{n}}$$

**Table 1:** The calculated error between $\:{x}^{2}$and $\:{x}^{3}$ curves and their corresponding linear value.

Approximate equation	NMAE	NRMSE
$\:{x}^{2}$	0.0121	0.0183
$\:{x}^{3}$	0.0216	0.0468

The findings presented in Table 1 indicate a minimal error in the approximation of the $\:{x}^{2}$and $\:{x}^{3}$. However, to further explore the impact of this error, it is essential to examine time and phase response of the LHR model compared to HR neuron. In this study, the learning process is characterized by spiking burst behavior, leading to the assignment of model constants as $\:a=1,\:b=3,\:c=1,\:d=5,\:r=0.001,\:s=4,\:q=-1.618$. This configuration ensures that the burst behavior is evident in reaction to the input current received by the neuron.

**Fig. 2:**
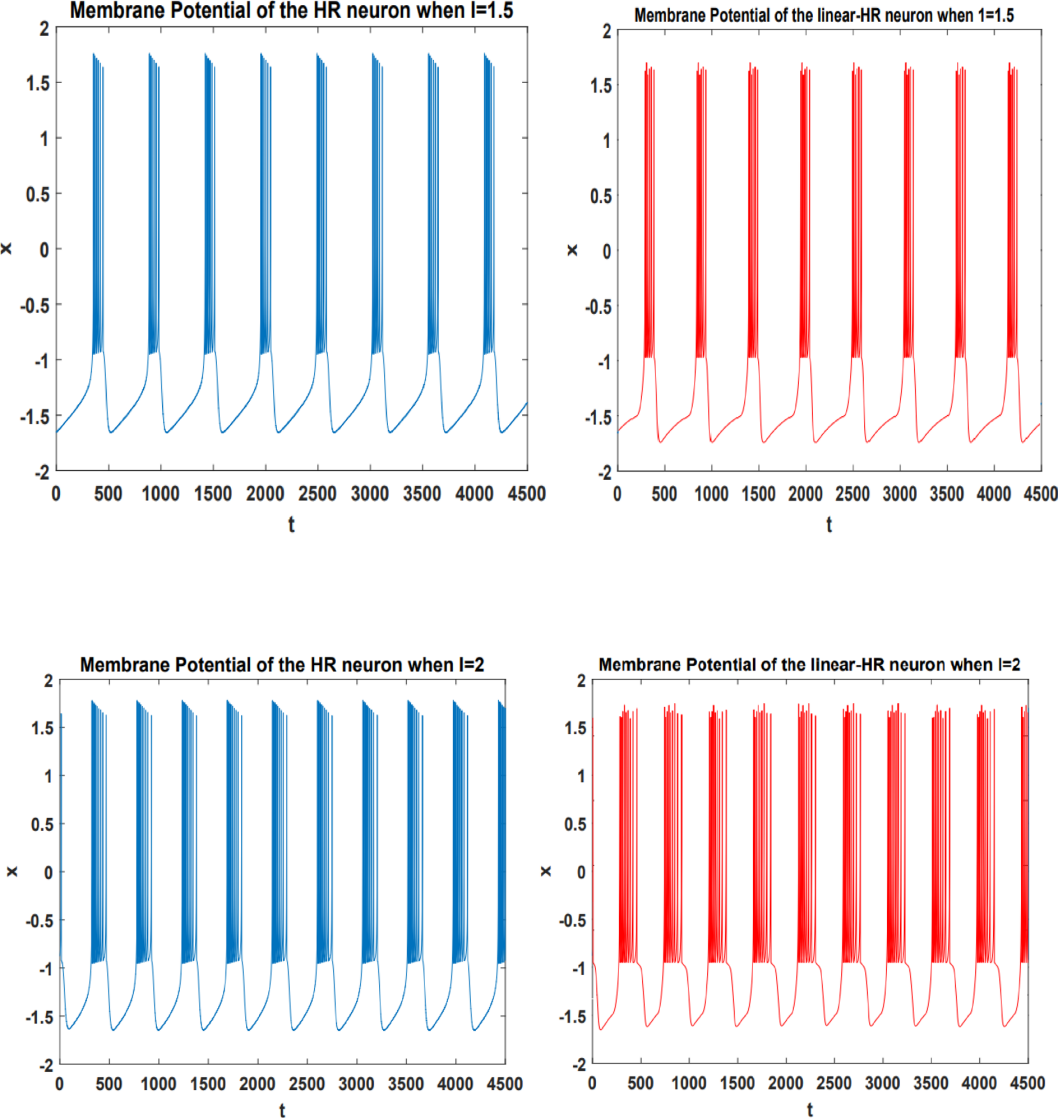
The burst activity of the HR and LHR neuron models in response to varying input currents.

The precision of the suggested LHR model aligns with that of the initial model during the burst firing activity, is illustrated in Fig. 2. This figure displays the membrane potential of both the HR and LHR neuron models in response to different input currents.

To assess the correlation between the firing activity pattern in the HR neuron (as described in Eq. 1) and the LHR model (outlined in Eq. 4), four error criteria were introduced, represented by Eq. 7 through 10.

7 $$\:\text{N}\text{o}\text{r}\text{m}\text{a}\text{l}\text{i}\text{z}\text{e}\text{d}\:\text{M}\text{e}\text{a}\text{n}\:\text{A}\text{b}\text{s}\text{o}\text{l}\text{u}\text{t}\text{e}\:\text{E}\text{r}\text{r}\text{o}\text{r}\:\left(\text{N}\text{M}\text{A}\text{E}\right)=\frac{1}{n}\sum\:_{i=1}^{n}\left|\frac{{R}_{\text{H}\text{R}}-{R}_{LinearHR}}{{Max}_{{R}_{\text{H}\text{R}}}}\right|$$

8 $$\:\text{C}\text{o}\text{r}\text{r}\text{e}\text{l}\text{a}\text{t}\text{i}\text{o}\text{n}=\frac{cov({R}_{\text{H}\text{R}},{R}_{L\text{H}\text{R}})}{{\sigma\:}_{{R}_{\text{H}\text{R}}}{\sigma\:}_{{R}_{L\text{H}\text{R}}}}$$

9 $$\:\text{N}\text{o}\text{r}\text{m}\text{a}\text{l}\text{i}\text{z}\text{e}\text{d}\:\text{R}\text{o}\text{o}\text{t}\:\text{M}\text{e}\text{a}\text{n}\:\text{S}\text{q}\text{u}\text{a}\text{r}\text{e}\:\text{E}\text{r}\text{r}\text{o}\text{r}\:\left(\text{N}\text{R}\text{M}\text{S}\text{E}\right)=\sqrt{\frac{\sum\:_{i=1}^{n}{\left(\frac{{R}_{\text{H}\text{R}}-{R}_{Linear\text{H}\text{R}}}{{Max}_{{R}_{\text{H}\text{R}}}}\right)}^{2}}{n}}$$

10 $$\:\text{N}\text{o}\text{r}\text{m}\text{a}\text{l}\text{i}\text{z}\text{e}\text{d}\:\text{F}\text{r}\text{e}\text{q}\text{u}\text{e}\text{n}\text{c}\text{y}\:\text{D}\text{i}\text{f}\text{f}\text{e}\text{r}\text{e}\text{n}\text{c}\text{e}\:\left(\text{N}\text{F}\text{D}\right)=\frac{\left|Mean\:of\:frequnce\text{H}\text{R}\:response-Mean\:of\:frequnce\:linearHR\:response\right|}{Maximum\:frequence\:\text{H}\text{R}\:response}$$

In Eqs. 7–10, the variables R_HR and R-LHR correspond to variable $\:x$ in the LHR and LHR model, respectively. The error metrics reported include the mean absolute error (NMAE), mean square error (NRMSE), correlation, and the differences in the spike and burst firing frequency (NFD-spike and NFD-burst). These metrics are summarized in Table 2, which presents the difference of LHR and HR model when input current is 1.5 and 2.

**Table 2:** Assessing behavioral similarity of the LHR model in relation to the standard HR model.

	Constant input current	NMAE	$\:\text{C}\text{o}\text{r}\text{r}\text{e}\text{l}\text{a}\text{t}\text{i}\text{o}\text{n}$	NRMSE	NFD_spike	NFD_burst
This work	**I = 1.5**	0.065	0.794	0.172	0.002	0.09
	$\:\varvec{I}=2$	0.1	0.89	0.2	0.006	0.15
N-LUT_HR^32^	**I = 1.5**	0.23	0.99	2.02	–	–
N-LUT_HR^32^	$\:\varvec{I}=2$	0.13	0.97	0.25	–	–
CORDIC-HR^33^	**I = 1.5**	0.0714	0.7612	0.1856	–	–
CORDIC-HR^33^	$\:\varvec{I}=2$	0.1008	0.8702	0.2055	–	–

The results shown in Table 2 distinctly demonstrate that, in comparison to the N-LUT-HR and CORDIC-HR neurons, the presented LHR neuron closely aligns with the firing activity of the standard HR neuron model, with significantly reduced error. As outlined in the training approach section, inter-spike interval (ISI) plays a significant role in altering synaptic weights, particularly influencing $\:\eta\:$, while inter-burst interval (IBI) affects the exponential term. The findings presented in Table 2; Fig. 2 further validate the strong alignment in performance between the LHR and original HR neurons.

**Fig. 3:**
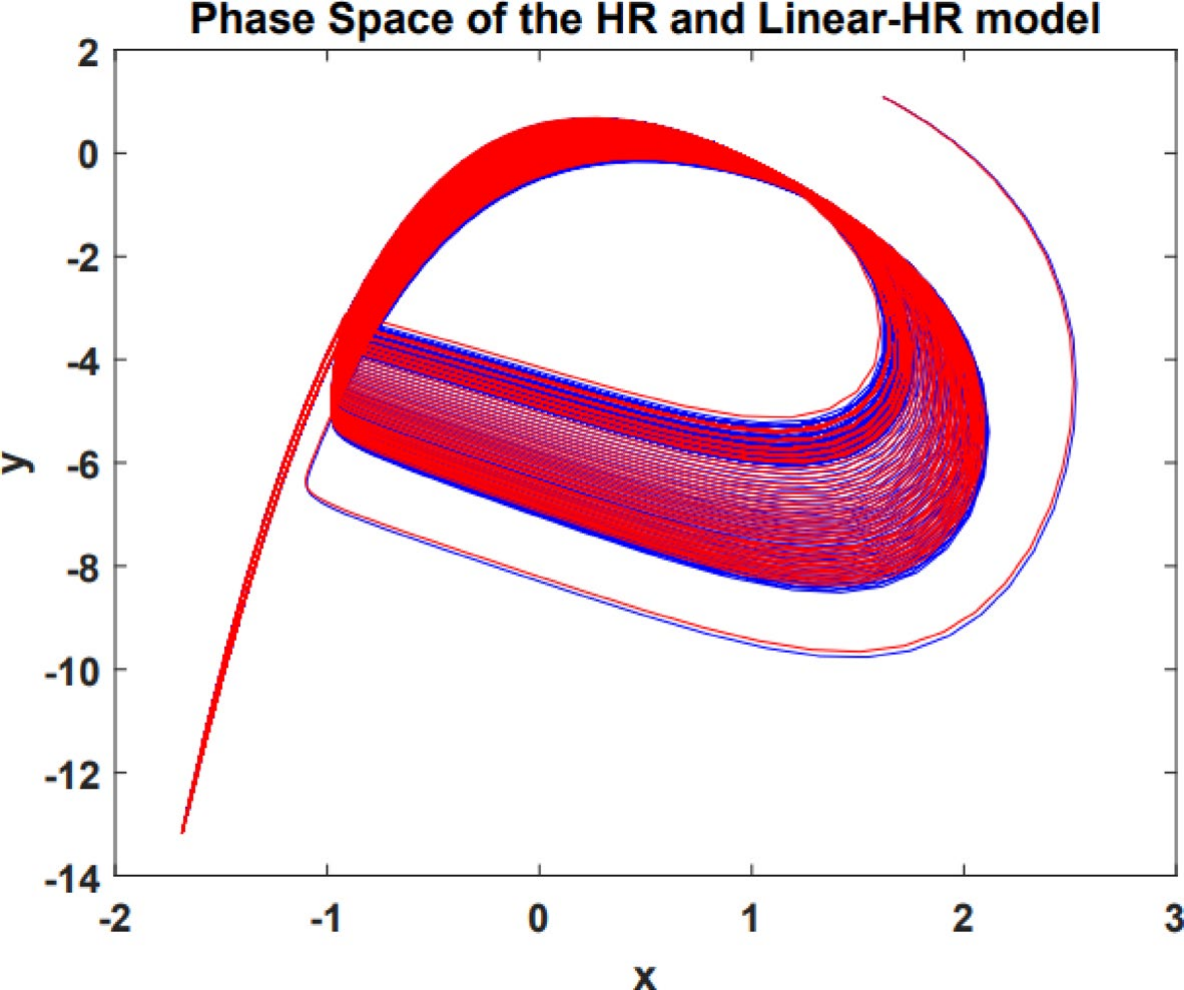
The phase behavior of the LHR model and HR model in red and blue, respectively.

The analysis of phase space serves as a crucial instrument to examine the dynamic analysis of systems. Analyzing the phase characteristics of the primary variables ($\:x,\:y$) in both the LHR and HR models can provide additional validation for the LHR model. Figure 3 illustrates the phase behavior of the LHR model and HR model in red and blue, respectively, while I = 1.5.

In summary, the assessments and comparisons of mean errors have illustrated the accuracy and effectiveness of the linear substitution method in simplifying the intricacies of non-linear functions within neurons. This approach can serve as a valuable and hardware-friendly method for the digital implementation of spiking pattern classification networks. Additionally, this linear substitution technique offers the advantage of facilitating the hardware design of $\:{x}^{2}$and $\:{x}^{3}$ functions through simple and low-cost digital modules likes shifter, add and sub operations, which consequently eliminating the requirement for a costly digital multiplier block.

In addition to the theoretical analysis and algorithm formulation conducted, it is essential to design and implement hardware to evaluate the efficacy of the suggested linear substitution method. The hardware digital design was implemented using the Virtex-7 XC7VX485T FPGA board. In the context of digital circuit design, it is essential to discretize all differential equations present in the proposed LHR model. Various discretization methods exist, including Runge-Kutta and Euler techniques; however, the first-order Euler method is preferred due to its simplicity and accuracy. The equations of LHR model that have been discretized are formulated as follows:

11 $$\:\left\{\begin{array}{l}x\left[n+1\right]=x\left[n\right]+\varDelta\:t\left(y\right[n]-a({a}_{1}x\left[n\right]+{b}_{1})+b({a}_{2}x\left[n\right]+{b}_{2})-Z[n]+I)\\\:y\left[n+1\right]=y\left[n\right]+\varDelta\:t(c-d\left({a}_{2}x\left[n\right]+{b}_{2}\right)-y\left[n\right])\\\:z\left[n+1\right]=z\left[n\right]+\varDelta\:t\left(r\left(s\left(x\left[n\right]-q\right)-z\left[n\right]\right)\right)\end{array}\right.$$

In Eq. 11, $\:\varDelta\:t$ represents the discretization step, which is set to 1/256 to facilitate a straightforward multiplication by a shift of 9 positions to the right. In the hardware design process, we aim to utilize shifting and addition as alternatives to multiplication for multiplying fixed parameters within variables. The numbers are utilized as fixed-point registers with the objective of minimizing hardware expenses. Due to the significant hardware overhead associated with floating point calculations, this design takes into account the minimum bit length while focusing on fixed point calculations.

**Fig. 4:**
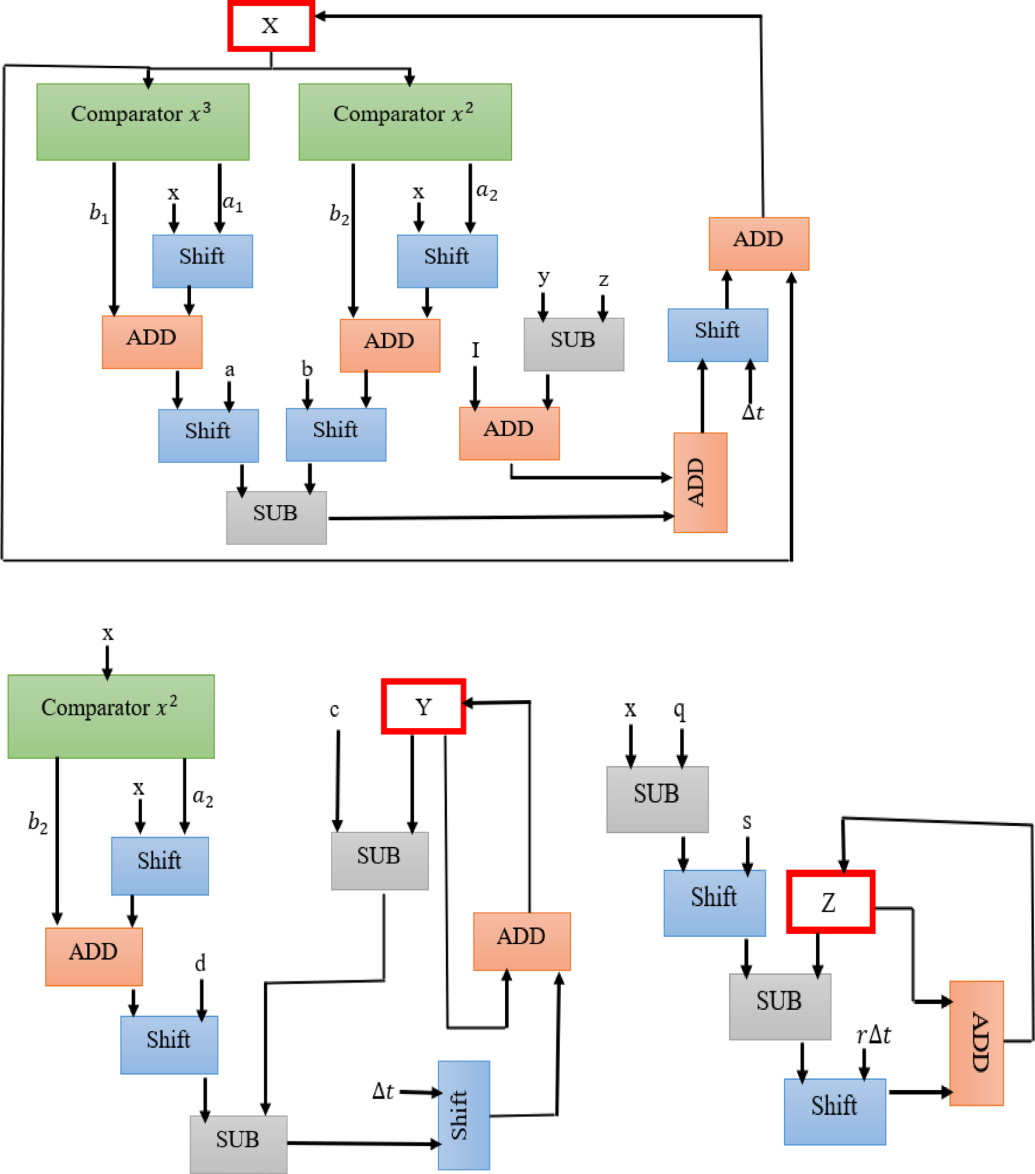
Hardware modules of the LHR neuron.

Figure 4 presents the hardware diagram for the LHR neuron. In this framework, expensive digital blocks are replaced with low-cost digital blocks such as comparators, shifts, and adders.

**Fig. 5:**
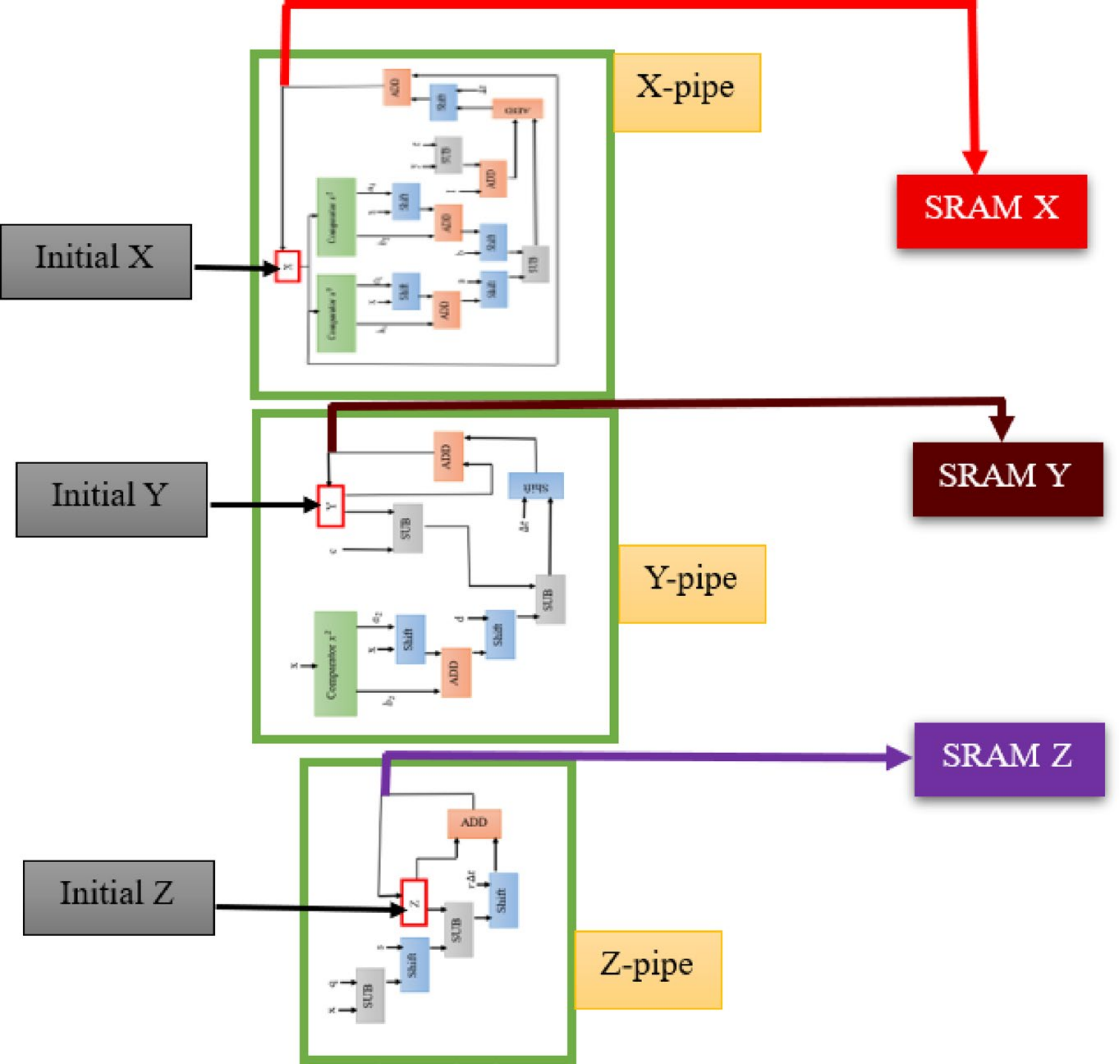
Comprehensive layout of the LHR digital circuit.

The overall configuration of the LHR digital module can be illustrated as shown in Fig. 5. As highlighted, the proposed LHR neuron model demonstrates a significantly lower error rate in comparison to the HR model, enabling a multiplier-less implementation in hardware. The hardware diagram indicates that the hardware circuit of the LHR neuron can only be achieved through the utilization of cost-effective adder, subtractor, and shift blocks.

The designs that were reviewed have been executed using VHDL on the Virtex 7 XC7 VX485T platform. The results of the hardware implementation for the proposed LHR neuron are presented in Table 3. Table 3 provide a comparison of the utilized hardware resource in the design of both the HR and LHR models. The findings indicated in Table 3 demonstrate that the LHR model exhibits minimal hardware expense and maximum frequency in comparison to the standard HR model and the other published hardware design. Given that the proposed LHR model demonstrates significantly lower error rates in replicating the functioning of the HR neuron across multiple dimensions in comparison to earlier studies, it presents a viable option for the implementation of spiking machine vision networks in hardware.

**Table 3:** The use of FPGA resources in the digital design of the hindmarsh Rose neuron.

Digital HR model	Slice Flip Flop	4-in-LUT	Speed	Power	Multiplier	Adder	Subtractor
Standard HR	852	268	25 MHz	180 mw	8	7	6
Digital HR model^34^	284	831	87.7 MHz	N/A	2	2	2
Digital modified biological Hindmarsh-Rose^35^	412	659	81.2 MHz	N/A	0	N/A	N/A
NC-PWL HR neuron^36^	469	N/A	139 MHz	110 mw	0	N/A	N/A
Digital HR model^37^	1173	2172	94.23 MHz	N/A	104	N/A	N/A
Jamshidi-HR-model^33^	285	224	110 MHz	N/A	0	4	3
This work (Virtex 7)	240	196	140 MHz	69 mw	0	3	3
This work (Virtex 2)	247	187	138 MHz	65 mw	0	3	3
This work (Zynq)	103	200	250 MHz	95 mw	0	3	3
This work (Spartan 6)	281	190	120 MHz	67 mw	0	3	3

In the hardware implementation of spiking networks, selecting a hardware approximation method that utilizes minimal hardware resources while preserving accuracy in spike responses is crucial, as hardware resources are finite, thereby restricting the capacity to implement a substantial number of neurons within the network. When comparing the CORDIC algorithm with the proposed method, it is essential to highlight that the CORDIC algorithm requires more hardware resources than our approach, as demonstrated in Table 3. However, the CORDIC algorithm excels in accurately reconstructing spiking behavior at higher iterations. Consequently, our proposed method is more advantageous than CORDIC for this particular application.

### Computational modeling of excitatory and inhibitory synapses

To achieve the objective of constructing a spiking network comprising both excitatory and inhibitory HR neurons, the synaptic interactions within the network will incorporate AMPA excitatory synapses and GABA inhibitory synapses.

The input current to neuron $\:k$ is composed of two components: $\:{I}_{AK}$, representing the AMPA excitatory neurotransmitter from the presynaptic excitatory neurons along with the input from spikes produced by the pseudo-retinal model. Additionally, the inhibitory input to neuron $\:k$ includes $\:{I}_{GK}\left(t\right)$, which denotes the GABA inhibitory current from the presynaptic inhibitory neurons^38^. The input current to neuron $\:k$ in Eq. 1 is generated by subtracting the total excitatory currents from the inhibitory currents, as follows:

12 $$\:I={I}_{AK}-{I}_{GK}$$

The excitatory synapse $\:{I}_{AK}$ responsible for delivering the excitatory input to neuron $\:k$ is characterized by Eq. 13 through the variable $\:{x}_{Ak}$.

13 $$\:{\tau\:}_{dA}\frac{{dI}_{Ak}}{dt}=-{I}_{Ak}+{x}_{Ak}$$

14 $$\:{\tau\:}_{rA}\frac{{dx}_{AK}}{dt}={-x}_{Ak}+{\tau\:}_{m}\left({J}_{k-Pyr}\sum\:_{pyr}\delta\:(t-{t}_{k-pyr}-{\tau\:}_{L})+{J}_{k-ext}\sum\:_{ext}\delta\:(t-{t}_{k-ext}-{\tau\:}_{L})\right)$$

Similarly, the inhibitory synapses $\:{I}_{GK}$ responsible for delivering the inhibitory input to neuron $\:k$ are characterized by Eq. 15 through the variable $\:{x}_{Gk}$.

15 $$\:{\tau\:}_{dG}\frac{{dI}_{Gk}}{dt}=-{I}_{Gk}+{x}_{Gk}$$

16 $$\:{\tau\:}_{rG}\frac{{dx}_{Gk}}{dt}={-x}_{Gk}+{\tau\:}_{m}\left({J}_{k-int}\sum\:_{int}\delta\:(t-{t}_{k-int}-{\tau\:}_{L})\right)$$

The value of parameters is reported in the Table 4^36^.

**Table 4:** The value of parameters of excitatory and inhibitory synaptic currents.

Parameter	Description	Value
$\:{\tau\:}_{m}$	Time constant of membrane potential	10 ms for interneurons and 20 ms for pyramidal neurons
$\:{\tau\:}_{rp}$	Time of refractory	2 ms/pyramidal neuron, 1 ms/interneuron
$\:{\tau\:}_{dA}$	Decay time of synapse (AMPA)	2 ms
$\:{\tau\:}_{rA}$	Rise time of synapse (AMPA)	0.4 ms
$\:{\tau\:}_{dG}$	Decay time of synapse (GABA)	5 ms
$\:{\tau\:}_{rG}$	Rise time of synapse (GABA)	1 ms
$\:{\tau\:}_{L}$	Latency of post-synaptic currents	1 ms
$\:{J}_{k-pyr}$	Weight of synapses between the neuron *k* and the excitatory neuron (pyramidal neuron, PY)	Initially chosen at random and adjust throughout the training process.
$\:{J}_{k-int}$	Weight of synapses between the neuron *k* and the inhibitory neuron (Interneuron, IN)	Initially chosen at random and adjust throughout the training process.

Consequently, four types of neural interactions can be defined as follows:

- Excitatory synaptic connectivity between excitatory neurons (PY) to other excitatory neurons (PY): PY to PY.
- Excitatory synaptic connectivity between excitatory neurons (PY) to inhibitory neurons (IN): PY to IN.
- Inhibitory synaptic connectivity between inhibitory neurons (IN) to excitatory neurons (PY): IN to PY.
- Inhibitory synaptic connectivity between inhibitory neurons (IN) to other inhibitory neurons (IN): IN to IN.

**Fig. 6:**
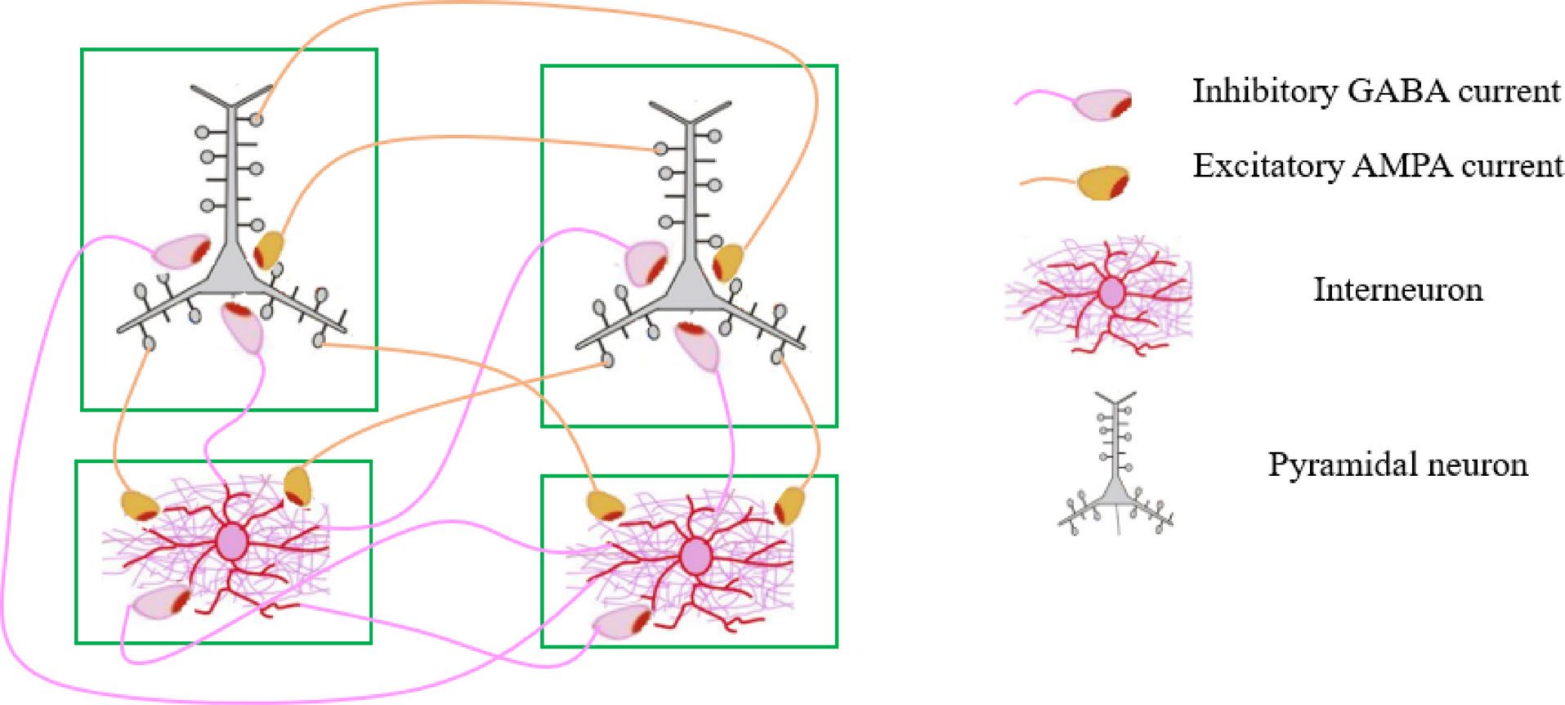
The type of synaptic connectivity between neuronal population within the spiking vision network encompasses four specific interactions: from pyramidal neurons (PY) to pyramidal neurons (PY), from pyramidal neurons (PY) to inhibitory neurons (IN), from inhibitory neurons (IN) to pyramidal neurons (PY), and from inhibitory neurons (IN) to inhibitory neurons (IN).

It is important to note that no learning takes place at the synaptic connectivity linking the input pseudo-retinal to the second layer’s neurons; rather, their values remain unchanged. Figure 6 illustrates the four identified synaptic connectivity that facilitate training process in the spiking vision networks. Interneurons serve to inhibit postsynaptic neurons, whereas pyramidal neurons function to excite them, as illustrated in Fig. 6.

## Configuration of spiking vision platform

This research presents three spiking vision networks (MNIST, CIFAR10, and CIFAR100 spike classification network) that share comparable learning mechanisms and structural designs, albeit with minor differences. This section analyzes the architecture of the four essential components that constitute spiking vision networks: the pseudo-retinal model as input layer, the large-scale neuronal population as second layer, the classifying neurons as output layer, and the Actor-Critic neuronal population.

### Pseudo-retinal model as input layer

**Fig. 7:**
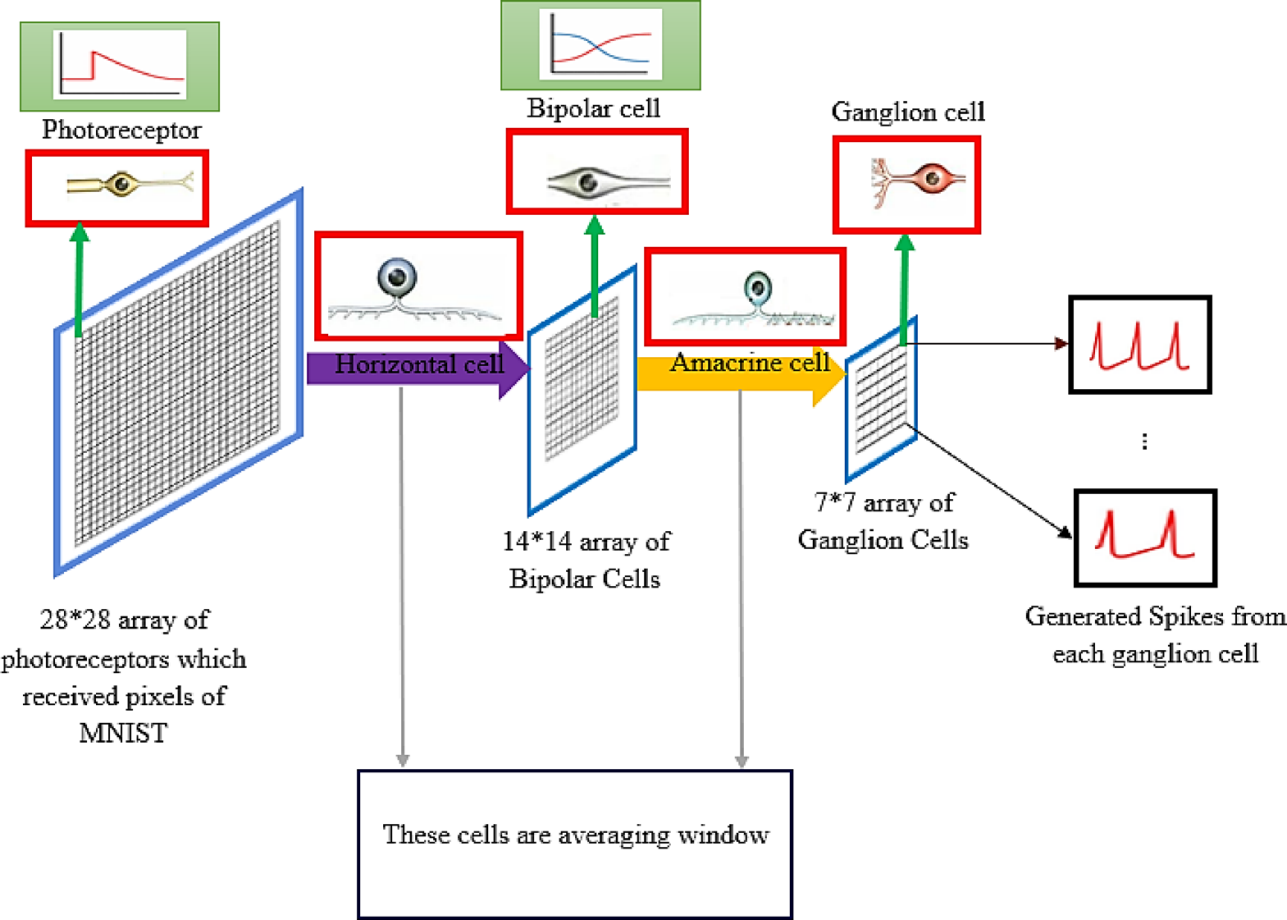
The pseudo-retinal model employed in the MNIST spike classification network transforms the input pattern of 28 by 28 pixels into a 7 by 7 spike train array.

The input layer of the spike classification network for MNIST, CIFAR10, and CIFAR100 utilizes the respective datasets of MNIST, CIFAR10, and CIFAR100. The MNIST dataset consists of handwritten digits (60000 samples/train and 10000 samples/test with 28 × 28 image pixels). In contrast, the CIFAR10 and CIFAR100 datasets are composed of 60,000 images, each with dimensions of 32 × 32 pixels, featuring natural images. Within these datasets, 50,000 images are designated for training purposes, while the remaining 10,000 images are reserved for testing. The input layer must convert the input data into spikes, before being injected into the spike classification network. The pseudo-retinal model, which is a computational model of the visual pathway, has been utilized to convert input image to spikes with reduced size. Figure 7 illustrates the pseudo-retinal model^39^ that was implemented in the spike vision network for classifying MNIST. Amacrine cells and horizontal networks have the capability to employ average windows to reduce the dimensions of the input pattern, while ganglion neurons serve as the origin of spikes. In the pseudo-retinal model of the MNIST spike classification network, the input image with pixels array 28*28 will be converted into a spike array 7 × 7, assuming that the averaging window of the amacrine and horizontal networks is set to 2 × 2 with a stride of 2. It is important to mention that the computational models for bipolar and ganglion cells are based on dynamic models referenced in sources^40^ and ^41^ respectively. In the retinal pathway illustrated in Fig. 7, the input image originates from the MNIST dataset. The output waveform from a cell within the 28 × 28 array of photoreceptors, as well as the output waveform from a cell in the 14 × 14 array of bipolar neurons, are depicted within green rectangles. Furthermore, within the 7 × 7 array, which consists of 49 output ganglion neurons, the spike train of two ganglion neurons is displayed at random (black rectangles). In reality, the output of the retinal pathway corresponding to the MNIST dataset comprises 49 spike trains, each with varying frequencies, with each spike train produced by a ganglion neuron situated in the 7 × 7 array.

Furthermore, the average window for the amacrine cells and horizontal networks is considered to be 2 × 2 with a stride of 2 in CIFAR10,100 spike classification network. In the pseudo-retinal model of the spike vision networks for classifying CIFAR10,100 patterns, ganglion cells produce a spike array of 8 × 8. The excitatory pyramidal and inhibitory interneuron in the second layer receive these spike trains, which are generated from input layers, at fixed weights that do not change during the learning process.

### large-scale neuronal population as second layer

The second layer of the spike vision networks for classifying MNIST and also CIFAR10 consists of 5,000 excitatory and inhibitory HR neurons, while the CIFAR100 network contains 10,000 neurons. Among these, 80% are excitatory pyramidal neurons, and 20% are inhibitory interneurons. In second layer, the neuronal interaction may exhibit full connectivity configuration. While spiking vision networks present a promising opportunity for decreased energy usage^1^, it is important to note that power usage escalates when considering slow learning process, the huge number of spikes, and full connectivity. Furthermore, earlier research has indicated that a fully connected configuration may impede the training speed and does not inherently improve the learning abilities of the network^42^. In second layer, a random connectivity pattern is selected, with the connection probability estimated at 0.2 based on biological data^43^. Specifically, the second layer of the spike vision networks for classifying MNIST, CIFAR10, and CIFAR100 consists of 5,010,650, 5,004,377, and 20,019,230 excitatory and inhibitory synapses.

The firing behavior of neural networks significantly influences their hardware energy consumption, highlighting the importance of sparse firing activity. In the second layer of spike vision networks, the sparse activity of neurons is amplified by incorporating a randomly synaptic connection between neurons.

In fact, as the physical separation between neurons grows, their degree of interaction decreases, which in turn leads to a reduction in the overall neuronal activity. The neurons in the second layer of spike vision networks are organized in a rectangular grid to emulate this interconnection effect. The connection strength between neurons diminishes as the distance increases, leading to the subsequent excitatory and inhibitory synapses Eq. 

17 $$\:{\tau\:}_{rA}\frac{{dx}_{AK}}{dt}={-x}_{Ak}+{\tau\:}_{m}\left({e}^{\frac{-r}{D}}{J}_{k-Pyr}\sum\:_{pyr}\delta\:(t-{t}_{k-pyr}-{\tau\:}_{L})+{J}_{k-ext}\sum\:_{ext}\delta\:(t-{t}_{k-ext}-{\tau\:}_{L})\right)$$

18 $$\:{\tau\:}_{rG}\frac{{dx}_{Gk}}{dt}={-x}_{Gk}+{{e}^{\frac{-r}{D}}\tau\:}_{m}\left({J}_{k-int}\sum\:_{int}\delta\:(t-{t}_{k-int}-{\tau\:}_{L})\right)$$

Where $\:r$ is the Euclidean distance between neurons which is scaled by $\:D$.

### Classifying neurons as output layer

The type of output layer neurons is excitatory pyramidal HR neuron. The equation governing the membrane potential of this layer neurons is presented in Eq. 1. If an excitatory neuron from the second layer excites the classifying neurons, Eqs. 13 and 14 are utilized. Conversely, if an inhibitory neuron from the second layer suppresses the classifying neurons, Eqs. 15 and 16 come into play. The neurons responsible for classification are located in the output layer of spike vision networks. Upon completion of the training process, the output layer of the spike vision networks for classifying MNIST, CIFAR10, and CIFAR100 comprises 10, 10, and 100 neurons. The second and third (output) layer are fully interconnected, with all classifying neurons being of the pyramidal type. Consequently, interactions of the types excitatory to excitatory (PY to PY) and inhibitory to excitatory (IN to PY) occur between the second and third layer. The initial 50 milliseconds of the simulation are disregarded, and the subsequent 2000 milliseconds are used to assess the firing (burst) rate of the neurons responsible for classification. A neuron in the classification layer is identified as the victorious neuron if it exhibits the highest firing rate over a 2000 milliseconds simulation period during the testing phase. The preceding section discussed the significance of limited firing activity within spike vision networks designed for pattern recognition. As a result, the neuron responsible for accurately classifying a pattern shows a firing rate of 6 bursts every 2000 milliseconds, while the other classifying neurons have a firing rate of 1 burst per 2000 milliseconds.

### Actor and critic neuronal population

In reinforcement training procedure, the Actor and Critic framework is frequently employed, especially in applications involving neural networks^44^. Within this framework, the critic evaluates the overall anticipated reward based on the environmental condition and the actor determines the appropriate actions to take in response. The RPE (reward prediction error) is represented by the $\:{\delta\:}_{t}$, which measures the extent to which the chosen response differs from the anticipated value. According to Eq. 19, $\:{\delta\:}_{t}\:$is determined by assessing the complete rewards obtained from the operation done, which is obtained from the difference of the current scaled state ($\:V\left({s}_{t+1}\right)$) from the previous state ($\:V\left({s}_{t}\right)$).

19 $$\:{\delta\:}_{t}={r}_{t+1}+\gamma\:V\left({s}_{t+1}\right)-V\left({s}_{t}\right)$$

The scaling factor for the state value is denoted as $\:\gamma\:=0.5$. A $\:\gamma\:$ value close to one suggests that future benefits are taken into account, whereas values approaching zero imply a focus on immediate rewards, with future rewards being largely overlooked. The calculation of RPE is crucial as it allows for the adjustment of the previous state value, thereby reducing the probability of errors and enhancing the chances of responses that lead to better outcomes.

Equation 20 is used to implement reinforcement training within neural networks. Previous studies have shown that a dopamine accumulation can operate in a manner akin to reward prediction error (RPE), as represented in Eq. 20.

20 $$\:D\left(t\right)=\dot{v}\left(t\right)+r\left(t\right)-\frac{1}{{\tau\:}_{r}}v\left(t\right)$$

In this context, the critic is depicted as a group of 20 h neurons and it’s firing rate utilized to ascertain the value of $\:v$ in Eq. 20. The reward from the environment and the scaling coefficient for the time constant are represented by $\:r$ and $\:{\tau\:}_{r}$, respectively. The critic population’s firing rate ($\:v$) in accordance with Eqs. 19 and 20 can be regarded as equivalent to the value of $\:V\left(S\right)$.

Additionally, 1,000 Poisson neurons is employed to simulate the reward prediction error. An excitatory connectivity by a delay of 1.5 milliseconds, along with an inhibitory connectivity by a delay of 25 milliseconds, connects the critic and RPE, thereby representing the term $\:\dot{v\left(t\right)}$^45^ in Eq. 20^46^. The output of the reinforcement learning block, referred to as the dopaminergic variable D(t), is derived from the model of the Actor and Critic populations. Consequently, the interactions between the critic and the reward prediction error neuronal populations, along with the rewards obtained from the environment, form the basis of the Actor and Critic circuits.

**Fig. 8:**
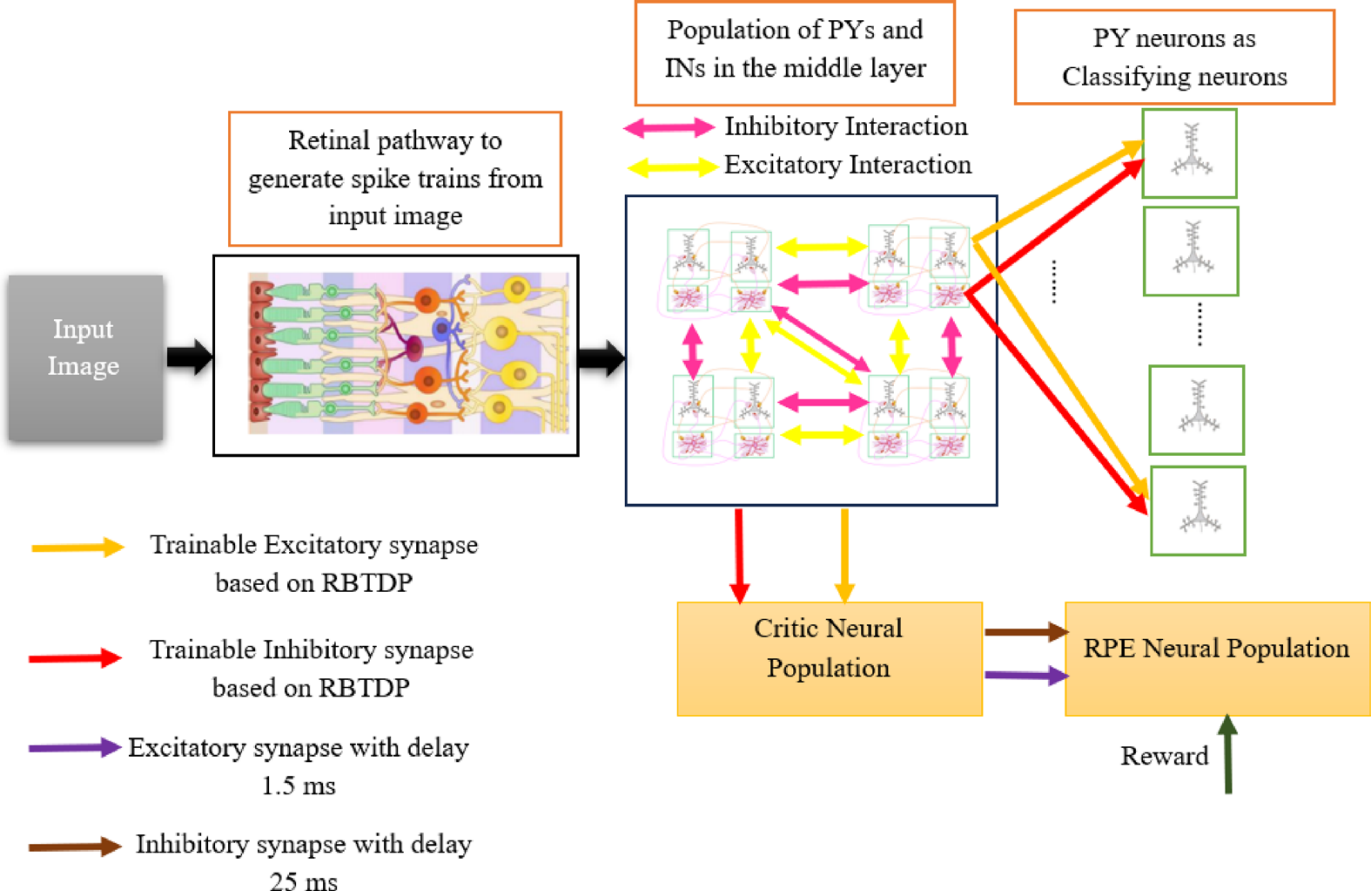
The overall design of the MNIST and CIFAR spike vision networks comprises several key components: a pseudo-retinal block serving as the input layer, a second and third layer, and both Actor and Critic neuronal modules. This architecture features modifiable synapses, specifically AMPA and GABA synapses in the second layer, which are optimized using BTDP. Additionally, excitatory and inhibitory synapses connecting the second layer to the third layer and Critic module are trained through RBTDP. Furthermore, the structure incorporates static synapses, which link the input layer to the second layer and connect the Critic module to the RPE neurons.

The training strategy outlined in this study (RBTDP) is derived from the outputs of both the Actor and Critic modules, integrated with BTDP learning. A comprehensive explanation of this will be provided in the subsequent section. The overall structure of the spike vision networks, comprising the pseudo-retinal input, the second layer, the third or output layer, and the Actor and Critic neuronal modules, is illustrated in Fig. 8.

## Reinforcement burst time dependent plasticity (RBTDP) learning

Recent studies have highlighted the significance of creating customized learning strategies in spiking networks, taking cues from the interactions among neurons in the brain. The advanced cognitive capabilities of the nervous system necessitate a harmonious integration of machine computation with neural spike computation to enhance machine learning performance^2^. Recent efforts have resulted in advancements in spiking networks that emulate the brain’s functional architecture^47^. These networks depend on the timing of neuronal spikes and the spatial connections among neurons to enhance the learning process^47^. The Hebbian and spike-timing-dependent plasticity (STDP) learning mechanisms are particularly noteworthy because of their biological relevance and their practical applications in SNNs. While supervised methods (spiking backpropagation)^21^ and the conversion of spike-based networks to their deep network equivalents^48^ exist, unsupervised STDP learning is highly valuable as it closely corresponds with biological findings. Changes in synaptic weight during spike-timing-dependent plasticity are influenced by LTP and LTD, dependent on the timing of spikes transmitted between neurons. Due to its effectiveness and widespread appeal, numerous variations of STDP, which fundamentally relies on unsupervised learning, have been developed.

Bursting behavior is thought to offer greater opportunities for information coding than individual spikes^17^. In this context, an unsupervised learning method known as Burst Time-Dependent Plasticity (BTDP), which is closely related to STDP, has been employed. This approach focuses on the encoding of information through time distance of spike (inter-spike intervals (ISI)) and time distance of burst (inter-burst intervals (IBI))^17^. The burst learning method aligns with biological research, where the update of synaptic weights occurs in relation to the burst timing of both postsynaptic and presynaptic neurons, until the training process is completed.

This paper subsequently introduces a novel update of the BTDP training mechanism by integrating BTDP with reinforcement learning. This altered mechanism is known as “Reinforcement-burst timing-dependent plasticity (RBTDP)” and affects the trainable excitatory and inhibitory synaptic connectivity.

Consequently, two distinct learning blocks are employed to train the proposed spiking network:

1. The synapses in the second layer are trained with the BTDP learning block.
2. The RBTDP learning block is utilized to train the synapses connecting the second layer to the third and critic layer. RBTDP combines elements of unsupervised learning (BTDP) with reinforcement learning, incorporating both Actor and Critic neural modules.

An unsupervised BTDP is employed to learn the weight of excitatory and inhibitory synaptic connectivity between neuron $\:i$ and neuron $\:j$ within the second layer. The changes in synaptic weights within the BTDP learning framework are outlined in Eq. 21 to 22^16^.

21 $$\:\varDelta\:{w}_{j}=\sum\:_{k=1}^{N}{\sum\:}_{l=1}^{N}W\left(IBI\right)$$

The term *IBI* refers to the time distance between burst activity of the post and pre-synaptic neurons. The function $\:W\left(x\right)$, is described as follows^16^:

22 $$\:W\left(x\right)=\left\{\begin{array}{c}{[A}_{+}+\frac{\sigma\:}{{\gamma\:}_{+}}]\text{exp}\left(\frac{-x}{\tau\:}\right)\:\:\:\:\:\:if\:x\ge\:0\\\:[{A}_{-}-\frac{\sigma\:}{{\gamma\:}_{-}}]\text{exp}\left(\frac{x}{\tau\:}\right)\:\:\:otherwise\end{array}\right.$$

$$\:\sigma\:=Average\:on\:ISI\:of\:post\:synaptic\:neuron\:\left({ISI}_{post}\right)-Average\:on\:ISI\:of\:pre\:synaptic\:neuron\left({ISI}_{pre}\right)$$.

Where $\:x$ equals $\:IBI$. According to Eq. 22, the alteration in weight of synapses occurs exponentially, with $\:\tau\:=10$ and adaptive learning rate represented as $\:{[A}_{+}+\frac{\sigma\:}{{\gamma\:}_{+}}]$ and $\:\left[{A}_{-}-\frac{\sigma\:}{{\gamma\:}_{-}}\right]$, which depend on the interspike interval (ISI) of both the postsynaptic and presynaptic neurons. In Eq. 22, the parameters are defined as follows: $\:{A}_{+}$ equals 0.07, $\:{A}_{-}$ equals − 0.05, $\:{\gamma\:}_{+}$ is 20, and $\:{\gamma\:}_{-}$ is 25. The value of $\:\sigma\:$ is determined using the time distance of spikes from post synaptic neuron ($\:{ISI}_{post}$) and the time distance of spikes from pre synaptic neuron ($\:{ISI}_{pre}$) in accordance with Eq. 22, resulting in an adaptive learning rate. The BTDP learning method is fundamentally grounded in STDP; however, it focuses on burst activities rather than spike activities. Additionally, it utilizes the time distance of bursts between pre- and post-synaptic neurons to establish an adaptive learning rate.

The RBTDP learning block represents a generalized form of BTDP learning, incorporating the influence of the dopaminergic $\:D\left(t\right)$, which is the output from the Actor and Critic neural modules, in the modification of synaptic weights. The framework for RBTDP learning is established according to Eq. 23.

23 $$\:\varDelta\:w=(D-{b}_{D})\varDelta\:{w}_{j}=(D-{b}_{D})\sum\:_{k=1}^{N}{\sum\:}_{l=1}^{N}W\left(IBI\right)$$

The synaptic connectivity between the second and third layer, or critic module, are adjusted according to the RBTDP learning mechanism. The parameter $\:{b}_{D}$ represents the reference value of the dopaminergic variable $\:D\left(t\right)$. It is determined using an average window of 10 milliseconds.

The utilization of learning blocks RBTDP and BTDP in synaptic formulation is crucial. On the other hand, recent research has underscored the importance of GABA-type and AMPA-type neurotransmitter transmission during the learning process. Consequently, the training rule has been integrated into the excitatory and inhibitory synaptic equations. Therefore, the training rules for the excitatory neurons of the second layer, which are considered based on BTDP, are expressed as follows:

24 $$\begin{aligned} & {\tau\:}_{rA}\frac{{dx}_{AK}}{dt}={-x}_{Ak}+{\tau\:}_{m}\left({e}^{\frac{-r}{D}}\left({J}_{k-Pyr}+\sum\:_{k=1}^{N}{\sum\:}_{l=1}^{N}(\stackrel{\sim}{A}\text{e}\text{x}\text{p}(-\frac{IBI}{\tau\:}\left)\right)\right) \right. \\ & \quad \left. \sum \:_{pyr}\delta\:(t-{t}_{k-pyr}-{\tau\:}_{L})+{J}_{k-ext}\sum\:_{ext}\delta\:(t-{t}_{k-ext}-{\tau\:}_{L})\right) \\ &\stackrel{\sim}{A}=\left\{\begin{array}{c}{[A}_{+}+\frac{\sigma\:}{{\gamma\:}_{+}}]\:\:\:\:\:\:if\:\:\:\:\:IBI > 0\\\:{[A}_{-}+\frac{\sigma\:}{{\gamma\:}_{-}}]\:\:\:\:\:\:if\:\:\:\:\:\:IBI < 0\end{array}\right.\end{aligned}$$

25 $$\begin{aligned} & {\tau\:}_{rG}\frac{{dx}_{AG}}{dt}={-x}_{AG}+{\tau\:}_{m}\left({e}^{\frac{-r}{D}}\left({J}_{k-int}+\sum\:_{k=1}^{N}{\sum\:}_{l=1}^{N}(\stackrel{\sim}{A}\text{e}\text{x}\text{p}(\frac{IBI}{\tau\:}\left)\right)\right)\sum\:_{int}\delta\:(t-{t}_{k-int}-{\tau\:}_{L})\right) \\ & \stackrel{\sim}{A}=\left\{\begin{array}{c}{[A}_{-}+\frac{\sigma\:}{{\gamma\:}_{-}}]\:\:\:\:\:\:if\:\:\:\:IBI>0\\\:{[A}_{+}+\frac{\sigma\:}{{\gamma\:}_{+}}]\:\:\:\:\:\:if\:\:\:\:\:\:IBI<0\end{array}\right.\end{aligned}$$

Furthermore, the training rules for the BTDP concerning the inhibitory neurons in the second layer are expressed as follows:

26 $$\begin{aligned} & {\tau\:}_{rA}\frac{{dx}_{AK}}{dt}={-x}_{Ak}+{\tau\:}_{m}\left({e}^{\frac{-r}{D}}\left({J}_{k-Pyr}+\sum\:_{k=1}^{N}{\sum\:}_{l=1}^{N}(\stackrel{\sim}{B}\text{e}\text{x}\text{p}(-\frac{IBI}{\tau\:}\left)\right)\right) \right. \\ & \quad \left. \sum\:_{pyr}\delta\:(t-{t}_{k-pyr}-{\tau\:}_{L})+{J}_{k-ext} \sum\:_{ext}\delta\:(t-{t}_{k-ext}-{\tau\:}_{L})\right)\\ & \stackrel{\sim}{B}=0.4*\left\{\begin{array}{c}{[A}_{+}+\frac{\sigma\:}{{\gamma\:}_{+}}]\:\:\:\:\:\:if\:\:\:\:\:IBI>0\\\:{[A}_{-}+\frac{\sigma\:}{{\gamma\:}_{-}}]\:\:\:\:\:\:if\:\:\:\:\:\:IBI<0\end{array}\right. \end{aligned}$$

$$\:{\tau\:}_{rG}\frac{{dx}_{AG}}{dt}={-x}_{AG}+{\tau\:}_{m}\left({e}^{\frac{-r}{D}}\left({J}_{k-int}+\sum\:_{k=1}^{N}{\sum\:}_{l=1}^{N}\left(\stackrel{\sim}{B}\text{exp}\left(\frac{IBI}{\tau\:}\right)\right)\right)\sum\:_{int}\delta\:(t-{t}_{k-int}-{\tau\:}_{L})\right)\:\:\:\:\:\:\:\:\:\:\:\:\:\:$$

27 $$\:\:\stackrel{\sim}{B}=0.4*\left\{\begin{array}{c}{[A}_{-}+\frac{\sigma\:}{{\gamma\:}_{-}}]\:\:\:\:\:\:if\:\:\:\:IBI>0\\\:{[A}_{+}+\frac{\sigma\:}{{\gamma\:}_{+}}]\:\:\:\:\:\:if\:\:\:\:IBI<0\end{array}\right.$$

Ultimately, the equations for RBTDP training pertaining to synapses linked to the third layer and the critic module are expressed as follows:

28 $$\begin{aligned} & {\tau\:}_{rA}\frac{{dx}_{AK}}{dt}={-x}_{Ak}+{\tau\:}_{m}\left({e}^{\frac{-r}{D}}\left({J}_{k-Pyr}+(D-{b}_{D})\sum\:_{k=1}^{N}{\sum\:}_{l=1}^{N}(\stackrel{\sim}{A}\text{e}\text{x}\text{p}(-\frac{IBI}{\tau\:}\left)\right)\right) \right. \\ & \quad \left. \sum\:_{pyr}\delta\:(t-{t}_{k-pyr}-{\tau\:}_{L})+{J}_{k-ext} \sum\:_{ext}\delta\:(t-{t}_{k-ext}-{\tau\:}_{L})\right) \\ & \stackrel{\sim}{A}=\left\{\begin{array}{c}{[A}_{+}+\frac{\sigma\:}{{\gamma\:}_{+}}]\:\:\:\:\:\:if\:\:\:\:\:IBI>0\\\:{[A}_{-}+\frac{\sigma\:}{{\gamma\:}_{-}}]\:\:\:\:\:\:if\:\:\:\:\:\:IBI<0\end{array}\right. \end{aligned}$$

29 $$\begin{aligned} & {\tau\:}_{rG}\frac{{dx}_{AG}}{dt}={-x}_{AG}+{\tau\:}_{m}\left({e}^{\frac{-r}{D}}\left({J}_{k-int}+(D-{b}_{D})\sum\:_{k=1}^{N}{\sum\:}_{l=1}^{N}(\stackrel{\sim}{A}\text{e}\text{x}\text{p}(\frac{IBI}{\tau\:}\left)\right)\right)\sum\:_{int}\delta\:(t-{t}_{k-int}-{\tau\:}_{L})\right) \\ & \stackrel{\sim}{A}=\left\{\begin{array}{c}{[A}_{-}+\frac{\sigma\:}{{\gamma\:}_{-}}]\:\:\:\:\:\:if\:\:\:\:IBI>0\\\:{[A}_{+}+\frac{\sigma\:}{{\gamma\:}_{+}}]\:\:\:\:\:\:if\:\:\:\:\:\:IBI<0\end{array}\right. \end{aligned}$$

The MNIST and CIFAR datasets were utilized for training and assessing spiking vision networks. To accurately evaluate the generalizability of the designed spike vision networks, the training and testing datasets are entirely distinct. The reported test accuracies are derived from five repetitions of tests conducted on the complete dataset. Before the commencement of the training phase, the synaptic weights were initialized using a normal distribution with a mean of 0 and a standard deviation of 0.2. In the training phase, input patterns are chosen at random from the training dataset. In the training phase, a 100-millisecond pause is implemented between two successive input patterns, which proves effective in achieving the equilibrium state of the dynamic equations. Throughout the learning, synaptic Eq. 24 to 29 are employed within the spiking vision networks. Conversely, throughout the test, weight updates are halted, and synaptic Eqs. 13, 15, 17, and 18 are utilized. The training period for spike vision networks to classify MNIST and CIFAR10 consists of 6 training sessions, while for CIFAR100, it extends to 30 training sessions. An increase in the number of training iterations is believed to have led to an improvement in accuracy exceeding 0.3%. A neuron that ceases its firing for 100 milliseconds in response to misclassification and then increases its firing rate twofold for an accurate classification emulates the dopamine variable $\:D$ utilized in the learning equations.

**Fig. 9:**
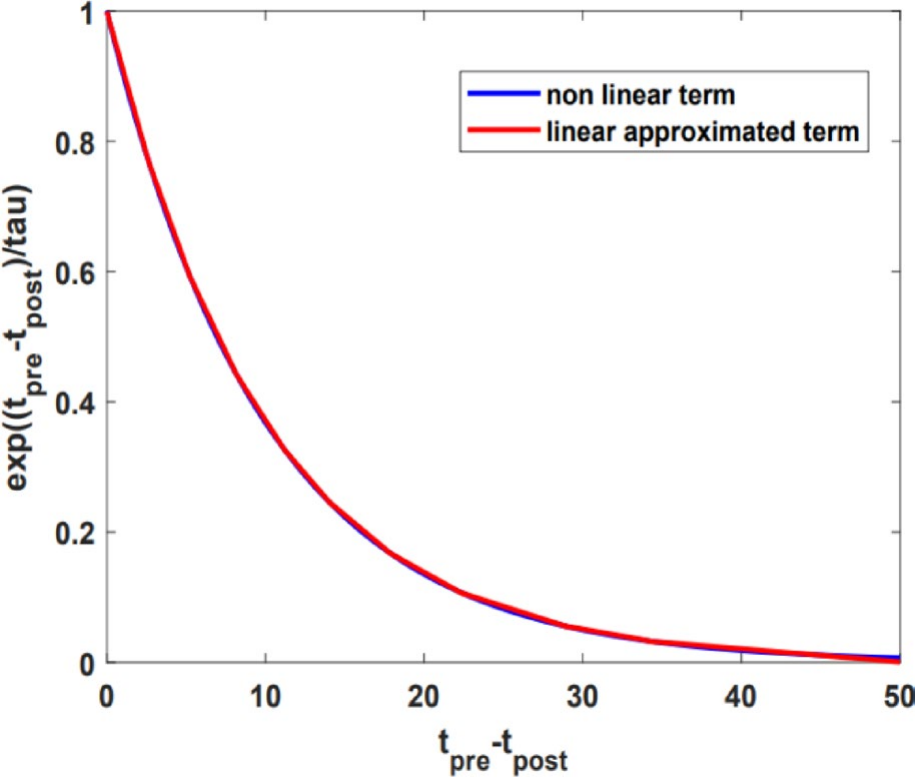
Linear approximation employed to simplify the exponential function within BTDP and RBTDP learning modules.

The nonlinear component in the BTDP and RBTDP learning equations is represented by an exponential function. Its linear approximation, as derived from the search algorithm discussed in Sect. 2, is illustrated in Fig. 9.

30 $$\:{\text{e}}^{\left(\frac{{t}_{pre\:}-{t}_{post\:}}{\tau\:}\right)}={a}_{3}({t}_{pre\:}-{t}_{post\:})+{b}_{3}=\left\{\begin{array}{l}-0.0876\:\times\:\left({t}_{pre\:}-{t}_{post\:}\right)+0.999\:\:\:\:\:if\:{t}_{pre\:}-{t}_{post\:}<2.63\\\:-0.0677\times\:\left({t}_{pre\:}-{t}_{post\:}\right)+0.9468\:\:\:\:\:\:\:if\:\:2.63<{t}_{pre\:}-{t}_{post\:}<5.23\\\:-0.0513\times\:\left({t}_{pre\:}-{t}_{post\:}\right)+0.8611\:\:\:\:\:\:\:\:\:\:\:\:if\:\:\:5.23<{t}_{pre\:}-{t}_{post\:}<8.19\\\:-0.0382\times\:\left({t}_{pre\:}-{t}_{post\:}\right)+0.7539\:\:\:\:\:\:\:if\:\:\:8.19<{t}_{pre\:}-{t}_{post\:}<11.12\:\:\:\\\:-0.0284\times\:\left({t}_{pre\:}-{t}_{post\:}\right)+0.6444\:\:\:\:\:\:\:\:\:\:\:\:\:\:if\:\:\:11.12<{t}_{pre\:}-{t}_{post\:}\:<14.15\\\:-0.0204\times\:\left({t}_{pre\:}-{t}_{post\:}\right)+0.5321\:\:\:\:\:\:\:\:\:\:\:\:\:if\:\:\:14.15<{t}_{pre\:}-{t}_{post\:}\:<17.71\\\:-0.0136\times\:\left({t}_{pre\:}-{t}_{post\:}\right)+0.4104\:\:\:if\:\:17.71<{t}_{pre\:}-{t}_{post\:}<22.43\\\:-0.0078\times\:({t}_{pre\:}-{t}_{post\:})+0.2810\:\:\:\:\:\:\:\:\:\:\:\:\:\:if\:\:\:22.43<{t}_{pre\:}-{t}_{post\:}\:<28.95\\\:-0.0043\times\:\left({t}_{pre\:}-{t}_{post\:}\right)+0.18\:\:\:\:\:\:\:\:\:\:\:\:if\:\:\:28.95<{t}_{pre\:}-{t}_{post\:}\:<34.17\\\:-0.0020\times\:\left({t}_{pre\:}-{t}_{post\:}\right)+0.1\:\:\:\:\:\:\:\:\:\:\:\:\:\:\:\:\:\:\:\:\:\:\:\:\:\:if\:\:\:\:{t}_{pre\:}-{t}_{post\:}>34.17\:\:\end{array}\right.$$

Table 5 presents the computed errors for the learning blocks utilizing both the exponential and linear approximations of the exponential function.

**Table 5:** The discrepancy criteria between the original and linear approximation learning modules.

Approximate equation	NMAE	NRMSE
BTDP and RBTDP	0.0024	0.0029

**Fig. 10:**
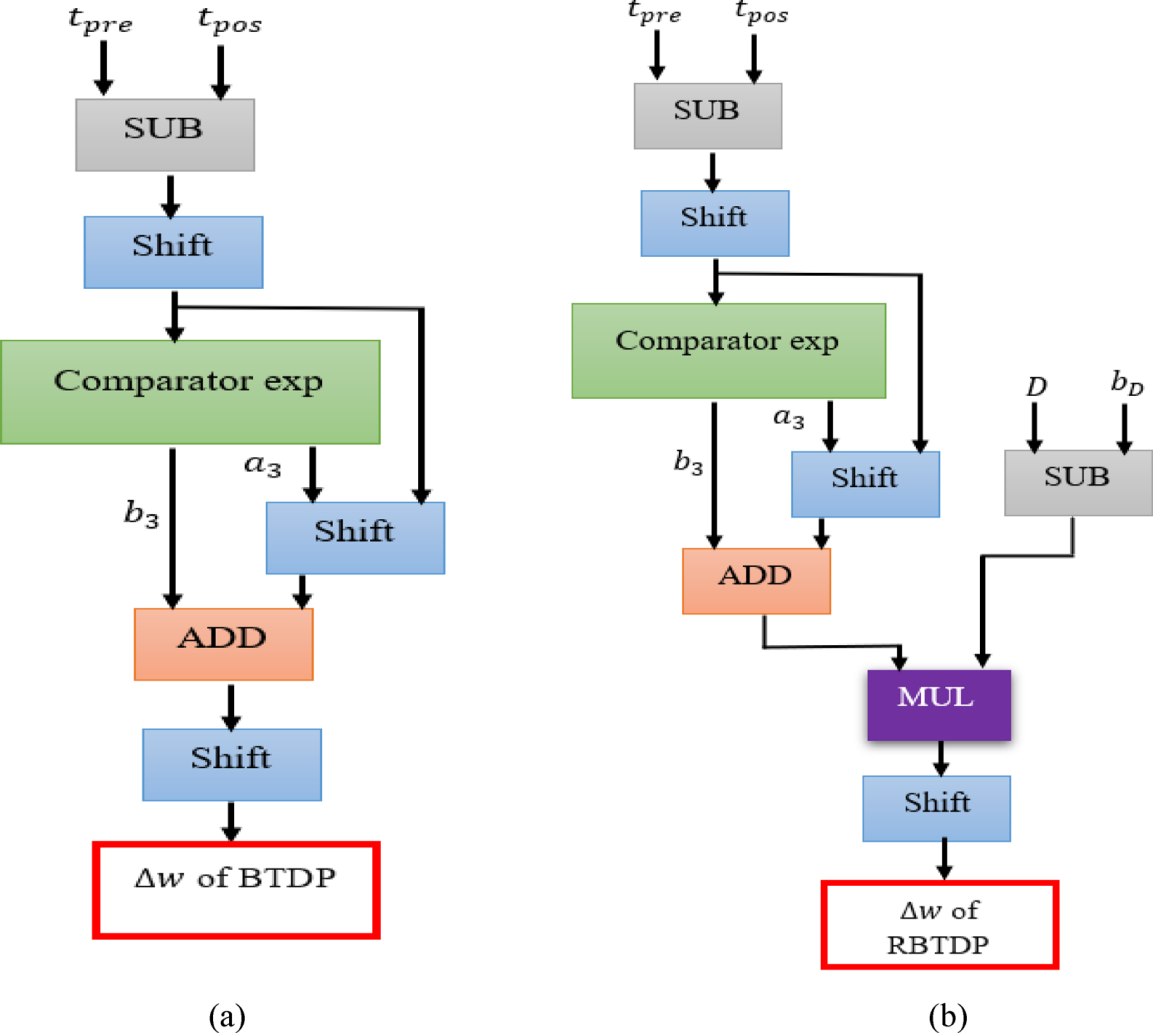
Hardware modules of (**a**) BTDP learning and (**b**) RBTDP learning module.

The results shown in Table 5 demonstrate that the error associated with the exponential term is minimal, which in turn leads to a low error in the learning blocks ($\:\varDelta\:w$). Additionally, as will be discussed in the following section, the learning digital module maintains both the accuracy and speed for the spike vision networks. Additionally, Fig. 10 illustrates the scheduling diagrams for the BTDP and RBTDP learning blocks.

The results concerning the utilization of FPGA resources in the hardware implementation of the spiking learning module are displayed in Table 6. The digital design of BTDP and RBTDP training modules employ a linear substitution of the exponential function, as illustrated in the Fig. 10.

**Table 6:** The use of FPGA resources in digital design of BTDP and RBTDP training modules.

Model	Slice flip flop	4-in-LUT	Speed	Power	Multiplier	Adder	Subtractor
BTDP	135	79	223 MHz	80 mw	0	2	1
RBTDP	173	148	201 MHz	72 mw	1	1	1

The digital circuits of BTDP and RBTDP learning blocks exhibit remarkable effectiveness in hardware computation solutions, characterized by a high frequency exceeding 200 MHz, minimal FPGA resource consumption, and an exceptionally low error. The average errors observed primarily stem from the intrinsic design features of the linear exponential within the digital learning modules, which can be effectively utilized in neural network platforms. Also, there is indeed a multiplier present in the learning block. Conversely, the implementation of a multiplier-free neuron holds significant importance, as neurons are the most commonly used components in constructing spike networks. Generally, while we require a learning block, it is crucial that this block prioritizes high performance accuracy over hardware resources, thereby justifying the use of a multiplier.

The foundation of the LHR neuron and the approximated learning module lies in accuracy in function computation, as will be detailed in the following section, while also remaining compatible with hardware requirements.

## Simulation results

Numerous spiking networks with machine vision application have been created in recent years. In comparison to earlier models, the suggested spike vision networks indicate improved accuracy, even with a reduced number of training epochs. This finding underscores the enhanced effectiveness of the suggested framework and training methodology. Tables 7 and 8, and 9 present a comparison of the test accuracies for the spike vision networks alongside various recently developed deep spiking neural networks.

At the end of Tables 7, 8 and 9, the hardware models utilized for the learning block and neurons in spike vision networks have been substituted, and the networks’ accuracy is displayed alongside the approximate hardware models. In contrast to several previously established spiking networks, the suggested spike vision networks, which include software model and its digital hardware counterpart, demonstrate improved classification accuracy while requiring fewer training epochs. Indeed, the subsequent tables adhered to two scenarios. In the first scenario, after the pattern recognition networks have undergone training and testing in software, with results documented in Tables 7, 8 and 9, the enhancement in the performance of the proposed networks compared to the best published work in recent years is clearly demonstrated as a result of the proposed learning.

In the second scenario, the digital neuron model and the digital learning module have taken the place of the original models. The pattern recognition networks have undergone retraining and evaluation, maintaining the same training and testing conditions as previously established to ensure a fair assessment can be conducted.

The outcomes of the second scenario, as presented in the final row of Tables 7, 8 and 9, indicate that pattern recognition networks utilizing digital neurons and learning via the proposed digital learning block can attain results that are comparable to those of the software model. Consequently, the suggested hardware (neuron and learning block) not only excels in terms of cost, power efficiency, and speed (as demonstrated in Tables 3 and 6), but also exhibits a high level of accuracy in adhering to the primary computational models utilized in machine vision applications.

**Table 7:** Comparison of performance of spike vision networks for MNIST classification.

Ref	Learning/architecture	Training type	Training accuracy	Training iteration
^49^	Self-Supervised learning	**-**	94.43	**-**
^50^	Evolutionary membrane learning algorithm	Supervised	90.8	**-**
^51^	Astrocyte-modulated plasticity	Unsupervised	96.15	Maximum of 5000 epochs
^52^	NAS framework to find best architecture	Supervised	93.2	–
^53^	M-LSM with multi-state fusion	Unsupervised	97.4	–
^54^	Training strategy with optimization	Unsupervised	93.6	–
^55^	Training method with threshold-driven	Supervised	96.8	28
^56^	Spiking Backpropagation learning	Supervised	97	500
This work	RBTDP	Unsupervised/reinforcement	98.2	6
	The Hardware RBTDP has been implemented on a spiking network utilizing LHR neurons.	Unsupervised/reinforcement	98.1	6

**Table 8:** Comparison of performance of spike vision networks for CIFAR10 classification.

Ref	Learning/architecture	Training type	Training accuracy	Training iteration
^57^	Spiking VGG and Residual network	Supervised	91.55	200
^58^	Spike backpropagation	Supervised	92.02	20
^21^	Spike backpropagation	Supervised	90.95	150
^59^	Training based on spike time	Supervised	93.49	20
^60^	Twin Network Augmentation (TNA) learning	Supervised	93.23	250
^61^	Training strategy with time of spikes	Supervised	93.54	200
^62^	Deep network to spiking network conversion	Supervised	93.16	–
^63^	Unsupervised STDP-based learning	Unsupervised	60	–
^64^	Spike activation lift training	Unsupervised	87.1	40
^65^	Active learning for a deep SNN model	Supervised	81.44	Maximum to 160
^66^	Training based on information dynamics with times	Supervised	92.04	120
^67^	Robust training for SNN	Supervised	92.54	100
^68^	Training feedback	Supervised	92.08	Maximum to 100
This work	RBTDP	Unsupervised/reinforcement	94	6
	The hardware RBTDP has been implemented on a spiking network utilizing LHR neurons	Unsupervised/reinforcement	93.8	6

**Table 9:** Comparison of performance of spike vision networks for CIFAR100 classification.

Ref	Learning/architecture	Training type	Training accuracy	Training iteration
^69^	Training with extreme quantization regime	Supervised	63.20	200
^70^	Gradient descent on DIET-SNN	Supervised	69.67	20
^59^	Training based on spike time	Supervised	71.05	20
^61^	Training strategy with time of spikes	Supervised	70.50	200
^62^	Deep network to spiking network conversion	Supervised	70.48	–
^71^	Training on synaptic weights and the membrane time constants	Supervised	74.36	600
^64^	Spike activation lift training	Unsupervised	59.11	40
^66^	Training based on information dynamics with times	Supervised	68.47	120
^72^	Cycle iteration learning	–	54.61	–
^68^	Training feedback	Supervised	64	Maximum 100 epochs
This work	RBTDP	Unsupervised/reinforcement	75.6	30
	The hardware RBTDP has been implemented on a spiking network utilizing LHR neuron	Unsupervised/reinforcement	75.25	30

The findings detailed in Tables 7, 8 and 9 are derived from the mean accuracy of 10 test executions of the MNIST, CIFAR10, and CIFAR100 detection networks across the complete test set, with standard deviations of 0.1, 0.23, and 0.3, respectively. The proposed network, utilizing unsupervised and reinforcement learning techniques, demonstrates superior accuracy in comparison to spiking vision networks, which are developed by converting deep to spiking counterparts. In the performance comparison tables for all the spiking networks presented, we indicated that the proposed learning method is a blend of unsupervised learning and reinforcement learning. It is crucial to note that the middle layer of the spiking networks progresses towards forming clusters based on the number of input data classes throughout the learning process, even in the absence of a defined cost function. As a result, the training in the middle layer occurs without supervision. The initial setup of the connections between the neurons in the output layer and those in the middle layer is characterized by a fully interconnected structure. During the learning process, each neuron in the output layer indeed forges a stronger connection with one of the clusters in the middle layer. Consequently, a cost function is not utilized in the output layer, leading to an unsupervised learning process that is executed through clustering. However, in the training of the output layer, since reinforcement learning is involved, and reinforcement learning operates based on the understanding of correct and incorrect responses, the learning of the output layer cannot be regarded as entirely unsupervised. The learning process for the middle layer is completely unsupervised, whereas the learning of the output layer represents a combination of methodologies. Given that no cost function was applied during the learning phase, we have classified the overall learning within the network as unsupervised learning, enhanced by reinforcement learning techniques.

**Fig. 11:**
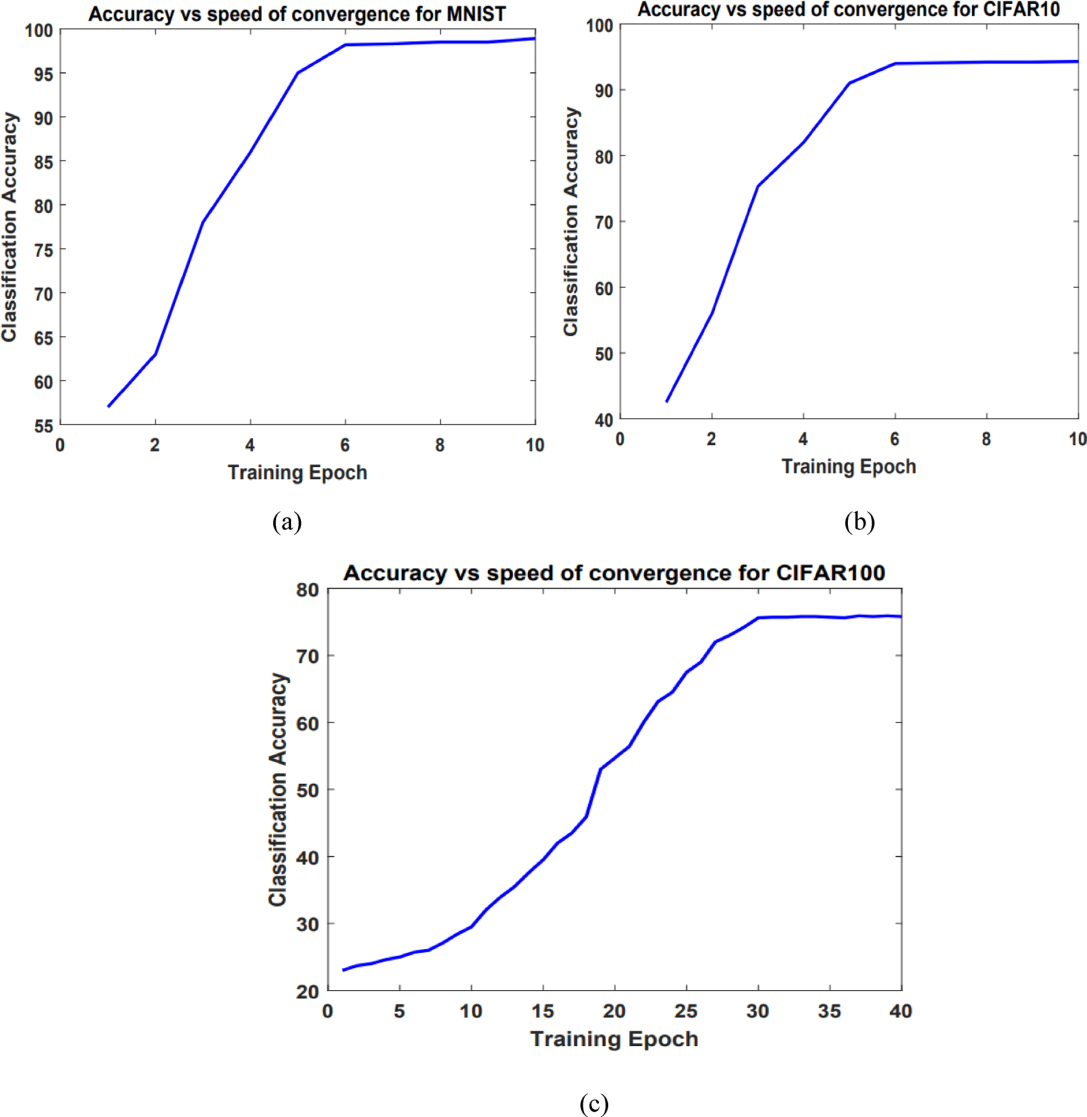
The relationship between the accuracy of classification and training iterations in the spike vision networks for classifying MNIST (**a**), CIFAR10 (**b**), and CIFAR100 (**c**).

In the proposed spiking vision networks, the training iterations are extended up to the enhancement in classification accuracy between two successive epochs drops below 0.3. Figure 11 illustrates how the variation in the training iterations affects the classification accuracy. Figure 11 shows that following iteration 6 in the spike vision networks for classifying MNIST and CIFAR10, as well as after iteration 30 in the CIFAR100 network, the classification accuracy shows fairly consistent, showing only slight enhancements.

Efforts were made to enhance the classification accuracy of spike vision networks for MNIST and CIFAR, by combining the RBTDP with reinforcement training. The objective of this integration was to attain classification accuracies of 98.2% for MNIST, 94% for CIFAR10, and 75.6% for CIFAR100. The accuracies attained by the proposed spike vision networks exceed those of previous spiking networks, including those that were trained in supervised settings. Although the accuracy of the proposed spiking networks does not match that of deep vision platforms, they excel in several areas, including unsupervised training, quicker convergence, and energy efficiently on neuromorphic hardware. Tables 7, 8 and 9 indicates the spiking vision networks training speed and their hardware counterparts, which necessitate a significantly lower training iterations in comparison to deep networks.

**Fig. 12:**
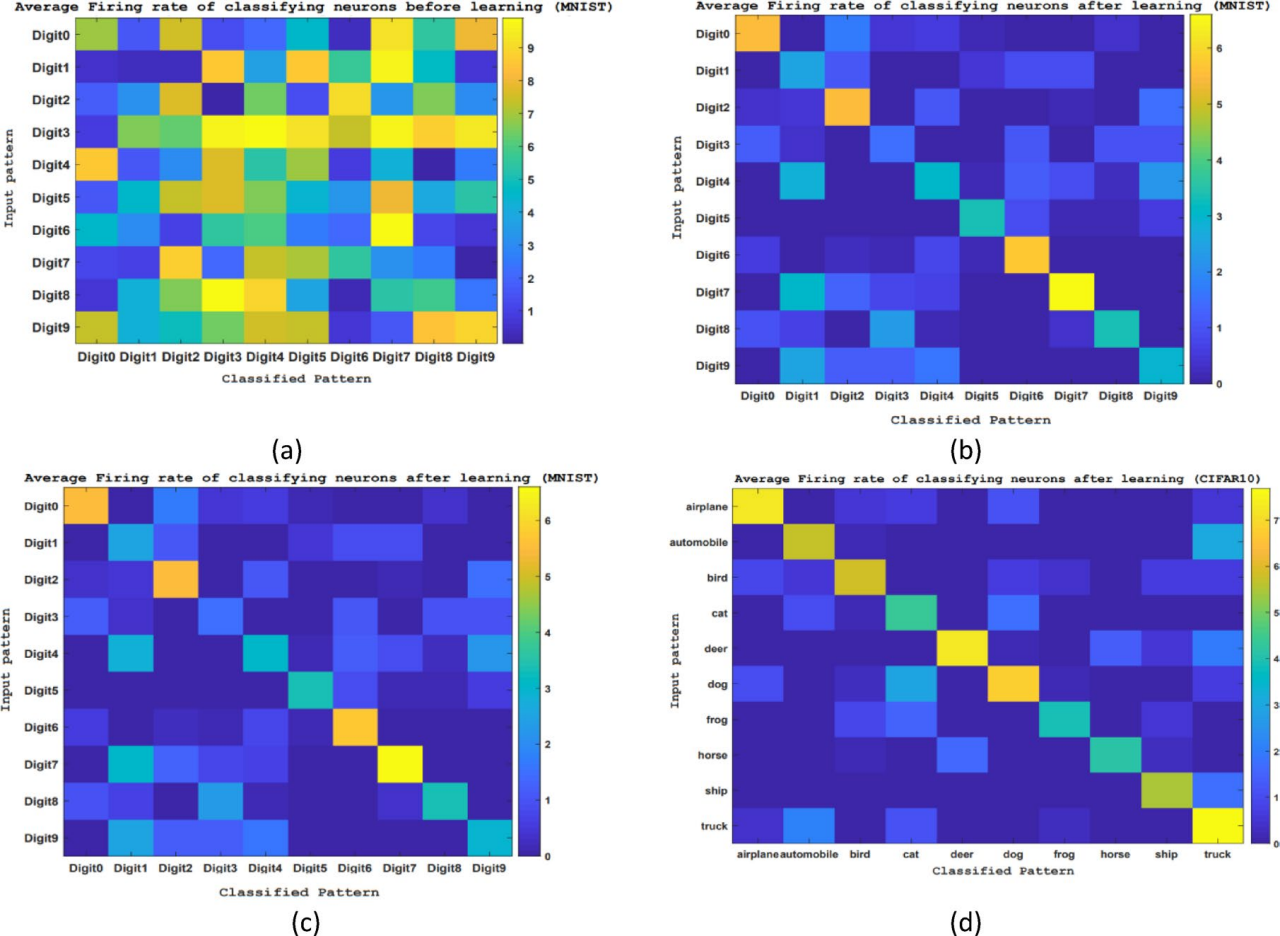
(**a**) and (**b**) represent the rates of burst activity in the output layer’s neurons when exposed to input patterns prior to learning. (**c**) and (**d**) illustrate the rates of burst activity in the output layer’s neurons in react to input patterns following the learning process.

Spiking networks offer a significant benefit compared to deep networks due to their considerably lower power usage. Recent developments in neuromorphic technology have facilitated the creation of spiking networks that function with low power usage on hardware. Spiking networks operate asynchronously and are driven by events, resulting in significantly lower power consumption compared to deep networks. Neuro-inspired chips utilize very little energy to send each spike throughout the network. Furthermore, by regulating the firing rate during both the learning and testing phases, the power consumption of spiking networks can be further reduced in comparison to other spiking platforms. This research aims to preserve sparse firing pattern within spike vision networks by eliminating full connectivity configuration and considering the impact of distance on neuronal interactions. The average firing (burst) rate of the classifying neurons is shown in Fig. 12; Nevertheless, the lack of available data makes it unfeasible to compare the firing activity rates of the proposed spiking vision networks with those of earlier spiking networks employed for comparable applications. The average burst rates for each classifying neuron are illustrated in Fig. 12(a) and (b) prior to the commencement of learning. Additionally, Fig. 12(c) and (d) display the average burst rates of the classifying neurons upon finishing the training process. Before training begins, the neurons responsible for classification in each of the spike vision networks display random reactions to input patterns, as demonstrated in Figs. 12(a-b). In contrast, once the training process is finished, these classifying neurons can effectively identify the input patterns with the highest rate of burst activity.

Specifically, the digits 3 and 4 represent a vulnerability in the MNIST classification spike network, which warrants further investigation in subsequent research. Nevertheless, the overall accuracy and convergence rate of the proposed spike network, in comparison to earlier studies, is clearly better in the findings presented in Tables 7, 8 and 9.

**Fig. 13:**
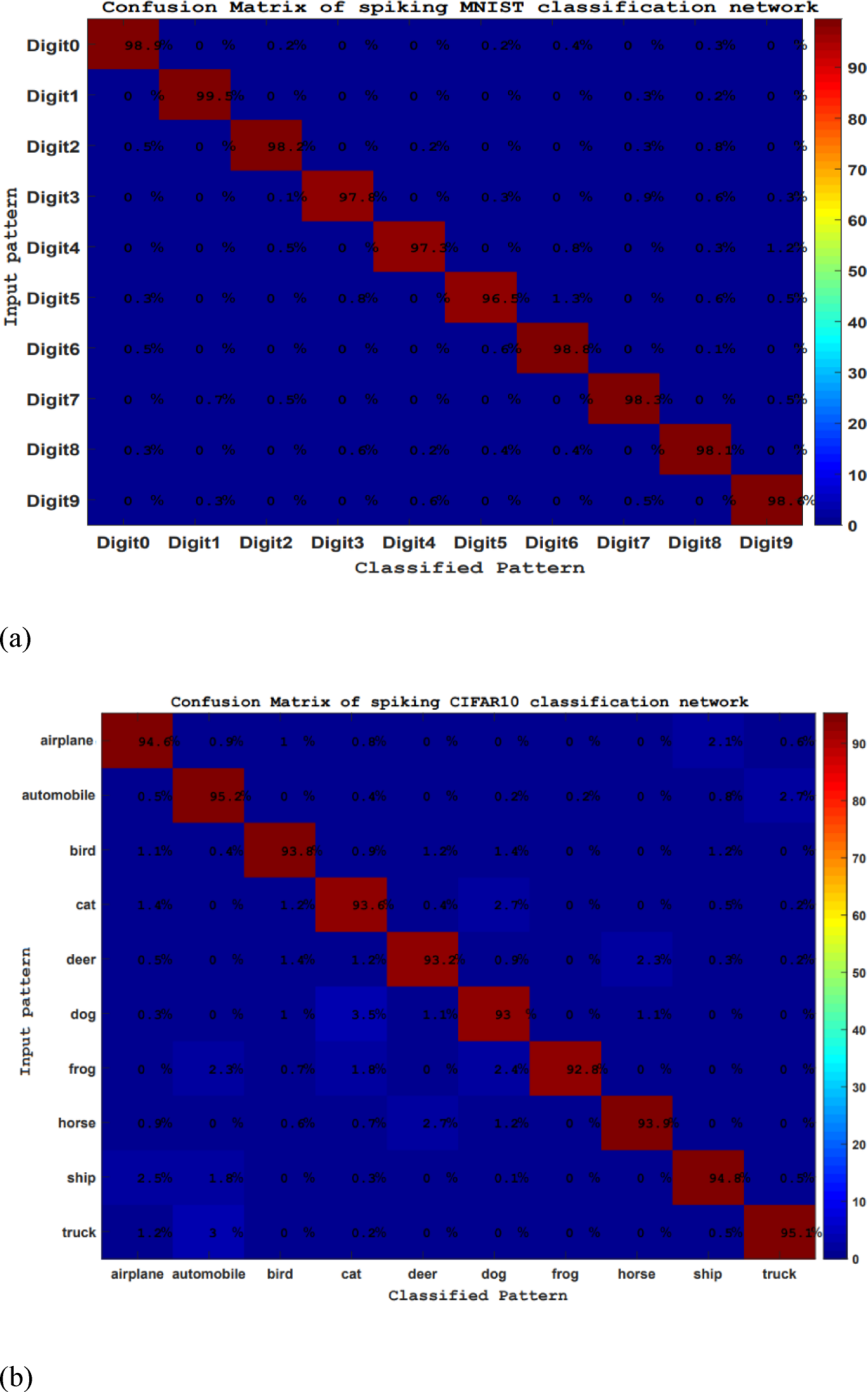
The confusion matrices for the spike MNIST recognition network (**a**) and the spike CIFAR10 recognition network (**b**).

To evaluate the accuracy of the spiking classifier in recognizing MNIST and CIFAR10 datasets more effectively, utilizing a confusion matrix is a suitable approach. The values along the diagonal of the confusion matrix indicate the proportion of correct classifications, while the other entries reflect the rate of misclassifications. The average value of each element along the diagonal of the confusion matrix corresponds to the recognition rate of the respective dataset. As illustrated in the confusion matrix presented in Fig. 13 for the proposed classification method applied to MNIST and CIFAR-10, the bio-inspired networks utilizing the suggested learning strategy demonstrate a high level of accuracy in pattern classification.

## Conclusion

This research showcases substantial advancements in spiking network applications through a methodology that incorporates a linear approximation of HR neurons and a learning module aimed at facilitating low-cost hardware design. Additionally, it features the proposed RBTDP learning approach and a bio-inspired model for spike image classification. The precise linear approximation method, which encompasses the Linear HR neuron and the linear learning block, demonstrates exceptional accuracy, elevated operational frequency, and efficient use of hardware resources, thereby validating their integration within neural network architectures. The effectiveness of the hardware design algorithms is validated through FPGA implementation, accompanied by an evaluation of performance that demonstrates minimum errors, low FPGA resource, and high frequency.

Initially, STDP learning was characterized as a common method of learning within the brain. Following this, the present paper introduces a novel modification of the STDP mechanism, referred to as “RBTDP.” The second and third layer, which includes the critic neural population, are trained using different learning mechanisms: BTDP for the second layer and RBTDP for the classification layer and critic neural module. These innovative training mechanisms have been incorporated into equation of synapses to develop training rules that capture the acknowledged impact of excitatory and inhibitory neurotransmitter on the training procedure. The proposed spike vision networks attained classification accuracy of 98.2% for MNIST, 94% for CIFAR10, and 75.6% for CIFAR100 with respectively, 6, 6, and 30 training iterations.

The results suggest that the presented spike vision networks exhibit distinct features, including sparse firing pattern throughout both train and test. These networks demonstrate enhanced accuracy in comparison to previous spiking vision networks, as well as a faster convergence rate than both spiking and deep vision networks. Furthermore, the classification accuracy achieved by the hardware model closely aligns with that of the original model, while the designed digital circuit is optimized for effective hardware implementation. The results validate that training strategies based on in biological findings, along with their corresponding hardware components, can serve as effective approaches for developing networks capable of recognizing patterns that require enhanced cognitive abilities.

## Data Availability

Data would be available through corresponding author with reasonable request.
